# The structure and substrate specificity of human Cdk12/Cyclin K

**DOI:** 10.1038/ncomms4505

**Published:** 2014-03-24

**Authors:** Christian A. Bösken, Lucas Farnung, Corinna Hintermair, Miriam Merzel Schachter, Karin Vogel-Bachmayr, Dalibor Blazek, Kanchan Anand, Robert P. Fisher, Dirk Eick, Matthias Geyer

**Affiliations:** 1 Group Physical Biochemistry, Center of Advanced European Studies and Research, Ludwig-Erhard-Allee 2, Bonn 53175, Germany; 2 Department of Physical Biochemistry, Max Planck Institute of Molecular Physiology, Otto-Hahn-Strasse 11, Dortmund 44227, Germany; 3 Department of Molecular Epigenetics, Helmholtz Center Munich, Center for Integrated Protein Science (CIPSM), Marchioninistrasse 25, München 81377, Germany; 4 Department of Structural and Chemical Biology, Icahn School of Medicine at Mount Sinai, New York, New York 10029, USA; 5 Central European Institute of Technology (CEITEC), Masaryk University, Brno 62500, Czech Republic

## Abstract

Phosphorylation of the RNA polymerase II C-terminal domain (CTD) by cyclin-dependent kinases is important for productive transcription. Here we determine the crystal structure of Cdk12/CycK and analyse its requirements for substrate recognition. Active Cdk12/CycK is arranged in an open conformation similar to that of Cdk9/CycT but different from those of cell cycle kinases. Cdk12 contains a C-terminal extension that folds onto the N- and C-terminal lobes thereby contacting the ATP ribose. The interaction is mediated by an HE motif followed by a polybasic cluster that is conserved in transcriptional CDKs. Cdk12/CycK showed the highest activity on a CTD substrate prephosphorylated at position Ser7, whereas the common Lys7 substitution was not recognized. Flavopiridol is most potent towards Cdk12 but was still 10-fold more potent towards Cdk9. T-loop phosphorylation of Cdk12 required coexpression with a Cdk-activating kinase. These results suggest the regulation of Pol II elongation by a relay of transcriptionally active CTD kinases.

## Abstract (web summary)

Cyclin-dependent kinase 12 (Cdk12) phosphorylates the C-terminal domain (CTD) of RNA polymerase II to regulate transcription. Here, the authors solve the crystal structure of the Cdk12 kinase domain and show that Cdk12 has its highest activity on a CTD substrate that carries a serine 7 phosphorylation.

## Graphical abstract

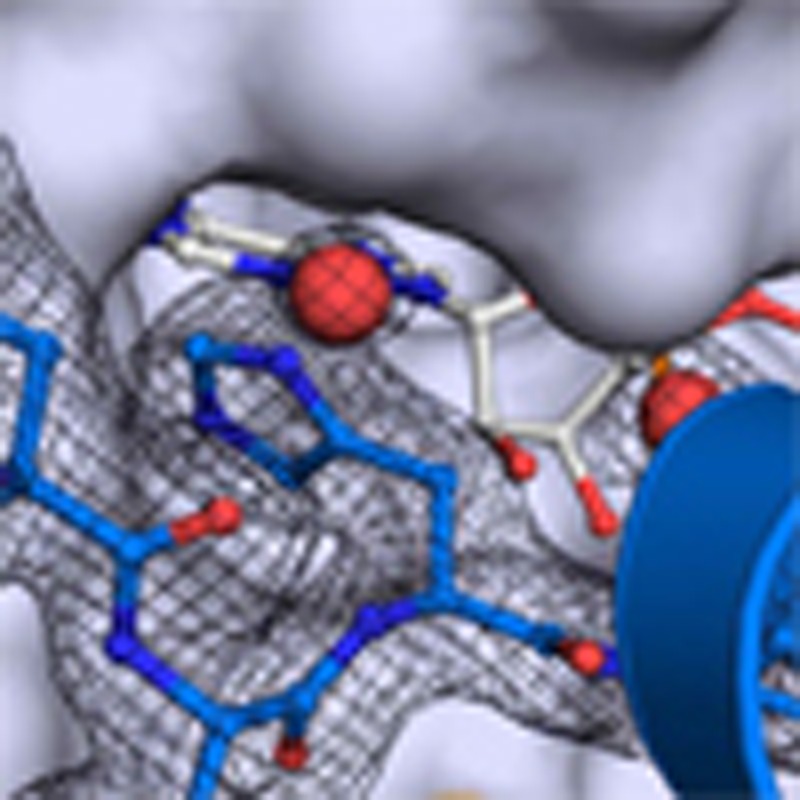

Cell division and transcription are tightly regulated by cyclin-dependent kinases (CDKs) and their regulatory cyclins. Whereas Cdk2/Cyclin A (CycA) and Cdk4/Cyclin D (CycD) are major players involved in the regulation of the cell cycle, five kinases—Cdk7, Cdk8, Cdk9, Cdk12 and Cdk13—have now been described as transcription-regulating kinases^1^. They phosphorylate the C-terminal domain (CTD) of RNA polymerase II (Pol II), thereby regulating different phases of the transcription cycle from transcription initiation to elongation and termination^2^. The CDKs and their corresponding cyclins form specific complexes including the Cdk7/CycH/Mat1 complex, which is part of the general transcription factor TFIIH, the Cdk8/CycC kinase module of the Mediator complex, the Cdk9/CycT complex that constitutes the active form of the positive transcription elongation factor (P-TEFb) and the recently discovered metazoan kinases Cdk12 and Cdk13, which both associate with Cyclin K (CycK).

The transcription-associated kinases phosphorylate serine residues within the hepta-repeat sequence Y_1_S_2_P_3_T_4_S_5_P_6_S_7_ of Rpb1, the largest subunit of RNA Pol II. The number of hepta-repeats varies from 26 in yeast to 52 in mammals, with some variation in the distal part of the CTD; among the variant repeats, ones with a lysine at position 7 are most common. The CTD is essential for cell viability, and partial truncations or site-specific mutations lead to specific growth defects^3^ and defects in recruitment of mRNA-processing machinery^4^. It is thought to act as a scaffold to coordinate the binding of proteins involved in the different phases of transcription and couples transcription with other nuclear processes such as mRNA maturation and the modification of chromatin. The three serines (Ser2, Ser5 and Ser7), the threonine (Thr4) and the tyrosine (Tyr1) of each repeat can be phosphorylated^5^,^6^. In addition, both peptidyl-prolyl bonds (Pro3 and Pro6) can be isomerized, the distal lysines were shown to be reversibly acetylated^7^ and an arginine at position 7 in a degenerate repeat can be methylated^8^. Combinations of these reversible CTD modifications spanning multiple repeats allow for a multitude of different states, which led to the hypothesis of a CTD code^9^.

The RNA Pol II transcription cycle starts with the recruitment of the unphosphorylated polymerase and the formation of the preinitiation complex. At the transcription start site (TSS), an increase in Ser7 and Ser5 phosphorylation is observed. While the signal for phosphorylated Ser7 (pSer7) remains high throughout the transcription cycle, the pSer5 signal decreases steadily towards the poly-adenylation (poly-A) site. Conversely, the content of pSer2 and pThr4 marks is low at the TSS but increases downstream. The signals for pSer2 are the highest at and downstream of the poly-A site, consistent with the recruitment of 3′-RNA processing factors by Ser2-phosphorylated CTD. High levels of pTyr1 marks in the body of genes in yeast instead favour the binding of elongation factors and prevent binding of termination factors to the CTD^10^. These phospho marks are removed by phosphatases during the termination process. It is a matter of debate whether the CTD phosphorylation cycle is uniform across all genes, or whether it is established in a gene-specific manner as suggested by two studies^11^,^12^. Another study showed that the phosphoserine marks are set and removed depending on their distance from the TSS and termination site, respectively, with no significant differences among genes^13^. A systematic approach examining the genome-wide distribution of CTD modifications indicated considerable interplay between CTD kinases and phosphatases, suggesting that transcription operates in a uniform mode at virtually all genes^14^.

The identification of Cdk12 and Cdk13 as CTD kinases in metazoans^15^,^16^,^17^ seemed to resolve a long-standing conundrum: the two yeast species *Saccharomyces cerevisiae* (*S.c.*) and *Schizosaccharomyces pombe* (*S.p.*) each have two CDKs required primarily during transcript elongation (Bur1 and Ctk1 in *S.c.*, and Cdk9 and Lsk1 in *S.p.*), whereas metazoans were thought to have only one (Cdk9). Whereas some Ser2 and Ser5 phosphorylation is ascribed to Bur1 and Cdk9 in budding and fission yeast, respectively; phosphorylation of Ser2 *in vivo* seems to be mostly due to the non-essential CDKs, Ctk1 in *S. cerevisiae* and Lsk1 in *S. pombe*^18^,^19^. Interspecies sequence alignments of kinase domains indicate that Bur1 and fission yeast Cdk9 are orthologues of metazoan Cdk9, whereas Ctk1 and Lsk1 are orthologues of metazoan Cdk12 and the closely related Cdk13. At 1,490 and 1,512 amino acids, respectively, human Cdk12 and Cdk13 are unusually large kinases that share 43% sequence identity and harbour a central kinase domain. The assignment of specific serine phosphorylations in the CTD to particular CDKs remains nonetheless uncertain, because different preferences were reported in different studies. Other kinase substrates such as the transcription elongation factor subunit Spt5 also contribute to the complexity^20^. Cdk7 prefers Ser5 as a substrate *in vitro*, as do its orthologues, budding yeast Kin28 and fission yeast Mcs6; however, Cdk7, Mcs6 and Kin28 have also been implicated in generating the pSer7 mark^21^,^22^,^23^,^24^.

We have determined the crystal structure of the human Cdk12/CycK pair in complex with ADP and aluminium fluoride as a transition state mimic at 2.2 Å resolution. We show that Cdk12 contains an additional C-terminal helix, αK, outside the canonical kinase fold that makes multiple water-mediated interactions with both the N- and C-terminal lobes as well as the ribose of the bound nucleotide. This helix, followed by a polybasic cluster, is conserved in CTD kinases regulating transcriptional elongation, and its importance was revealed by the gradual loss of kinase activity towards the CTD with progressive truncation of this C-terminal extension. Similar to most CDKs, Cdk12 requires T-loop phosphorylation by a CDK-activating kinase (CAK) for full activity. Finally, Cdk12 exhibits the highest activity on a CTD substrate prephosphorylated at Ser7 suggesting a kinase relay mechanism to order events during the transcription cycle.

## Results

### Structure of the Cdk12/Cyclin K complex

**Figure 1:**
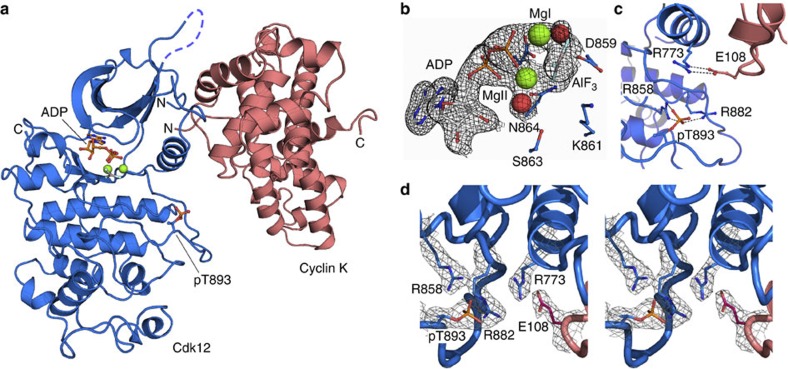
Structure of the human Cdk12/CycK complex. (**a**) Overall structure assembly of Cdk12 (blue) and CycK (red). The bound nucleotide and the phospho-threonine residue in the activation segment are highlighted. (**b**) Close-up of the kinase active site showing the ADP nucleotide, AlF_3_, Mg ions and water molecules. The final 2*F*_o_−*F*_c_ electron density is displayed at 1σ. (**c**) Close-up of pT893 in the Cdk12 T-loop segment and side chain contacts. R773 of the P_768_ITAIRE sequence forms electrostatic contacts with E108 of the K_105_VEE motif in CycK but is not directly contacting pT893. The other two canonical Cdk arginines, R858 and R882, form ionic interactions with the phosphate group. (**d**) Stereo image of the coordination of pT893 in the Cdk12 T-loop segment. The final 2*F*_o_−*F*_c_ electron density is displayed at 1σ.

Human Cdk12/CycK was expressed in baculovirus-infected insect cells co-transfected with Cak1 from *S. cerevisiae*, and the Cdk–cyclin complex was purified by affinity and size-exclusion chromatography (Supplementary Fig. 1). Crystals were grown in the presence of ADP, aluminium fluoride and the 13-residue substrate peptide P-pS-YSPTSP-pS-YSPT by the hanging drop diffusion technique. The structure was solved at a resolution of 2.2 Å by molecular replacement with the coordinates of CycK and Cdk9 as a search model (see Methods). The kinase complex was refined to an *R*_work_ of 19.5% and *R*_free_ of 24.0% with excellent stereochemistry (Table 1). Two Cdk12/CycK heterodimers form the asymmetrical unit cell of the protein crystal, whereas no crystallographic density was observed for the substrate peptide but only for the AlF_3_ transition state mimic (Fig. 1a,b). Overall, the structures of Cdk12 and CycK in the Cdk12/CycK complex are similar to previously determined structures of other CDKs and cyclins. However, there are substantial differences in the assembly of the two subunits and an unexpected conformation of the kinase C-terminal extension that associates with the ATP substrate. During revision of this manuscript, a crystal structure of Cdk12/CycK at 3.15 Å resolution under the accession number 4CJY was released from the Protein Data Bank.

The orientation of the cyclin with respect to the Cdk is rotated by about 25° compared with the cell cycle Cdk–cyclin complexes (for example, Cdk2/CycA; Supplementary Fig. 2). This arrangement is similar to that of Cdk9/CycT1 (ref. 25)^25^, but the buried molecular surface area of 2,294 Å^2^ upon Cdk12/CycK complex formation (corresponding to 79% of the Cdk2/CycA interface) is significantly larger than that of Cdk9/CycT1 (1,763 Å^2^, 61%). The Cdk12/CycK interface involves only the N-terminal lobe of the kinase; in contrast, Cdk2/CycA buried surface area involves both kinase lobes and encompasses 2,892 Å^2^. Cyclins involved in cell cycle control (CycA, CycB and CycE) and those involved in transcription (CycT1 and T2, CycC, CycH and CycK) vary significantly outside the classical cyclin box fold in length and orientation of the adjacent H_N_ and H_C_ helices. The gain in interface area in Cdk12/CycK compared with Cdk9/CycT1 is partly due to additional contacts of the N-terminal region of CycK (residues 19–22) with the N-terminal lobe of the kinase. Extensive electrostatic interactions are made between the second glutamate (E108) of the cyclin motif KΦEEΦ (where Φ is a hydrophobic residue) at the end of helix H3 and R773 of the PITAIRE helix motif of the kinase (Fig. 1c). In addition, R145 in helix H5 of CycK forms multiple interactions with the kinase N-terminal lobe, which are likely to contribute to the specificity of the Cdk12/CycK complex formation because this residue is not conserved in other cyclins (Supplementary Figs 3 and 4). The C-terminal region of CycK following the cyclin box fold is rich in prolines and glutamines and has been omitted for crystallization.

Interestingly, the ‘hydrophobic pocket’ present in CycA and CycE (characterized by the ^210^MRAILVDWxxE^220^ sequence in helix H1 of CycA), which serves as a recruitment site for Cdk2 substrates and the p27^Kip1^ inhibitor^26^,^27^, is replaced by the highly polar sequence ^49^YRREGARFxxD^59^ in CycK. In CycA, the acidic residue E220 following the MRAIL motif interacts with the arginine of the RxL recruitment motif in kinase substrates. This position corresponds to D59 in CycK and is accessible for possible interactions with regulatory factors (Supplementary Fig. 5). The same site in CycT1 is partly hidden by the presence of a smaller leucine (L44) compared with F56 in CycK and W217 in CycA. In addition, both CycT1 and CycK contain a long loop inserted between helices H4 and H5 (Supplementary Fig. 3). These differences could generate the recognition specificity for Cdk–Cyclin regulatory factors. Substrates, inhibitors and activators such as the Tat protein or Hexim1 were all shown to interact with this surface patch of the cyclin subunit in different Cdk–Cyclin complexes^26^,^27^,^28^,^29^,^30^.

### The kinase active center

The Cdk12 structure exhibits a typical kinase fold comprising the N-terminal lobe (715–816) and the C-terminal lobe (817–1,020) with 41.2% or 36.7% sequence identity to Cdk9 or Cdk2, respectively. Cdk12 is in the active kinase conformation as characterized by the orientation of the αC helix, also known as the PITAIRE helix (PITALRE in Cdk9; Supplementary Fig. 6). The ^877^DFG motif at the start of the activation segment and the activation segment itself adopt a conformation that allows access of the substrate to the catalytic site. Phospho-T893 within the activation segment is clearly visible in the electron density map and interacts with R858 and R882 (Fig. 1c). The third canonical arginine R773 is about 6.6 Å away but tightly interacts with the KΦEEΦ of CycK as seen in the stereo image of the electron density map (Fig. 1d).

The glycine-rich loop ^734^GEGTYG, or P-loop, which functions as a lid atop the ATP phosphates, coordinates the phosphates with the main chain amide group and the side chain hydroxyl group of T737 reaching out to the β-phosphate of the nucleotide. The presence of ADP and AlF_3_ in the crystallization condition led to the incorporation of ADP·AlF_3_ and two magnesium ions, MgI and MgII, in the crystal structure (Fig. 1b). The second magnesium ion MgII is coordinated in an almost ideal hexa-coordinate octahedral geometry with distances between 1.9 and 2.3 Å. It is coordinated by ADP α- and β-phosphate oxygens, D877 of the DFG motif, N864, one of the AlF_3_ fluorine atoms, and a single water molecule (Supplementary Fig. 7). The first magnesium MgI is observed in the same position as previously seen for protein kinase A and a Cdk2/CycA transition state complex^31^,^32^. MgI is less well coordinated and the occupancy in the crystal lattice appears reduced, which might go along with its proposed function as the catalytic Mg^2+^ (ref. 33). The catalytically important D859 points with its carboxyl side chain to AlF_3_ with a distance of 3.0 Å (Supplementary Fig. 7). The glycine-rich loop and the ATP transition state mimic is far from any crystallographic neighbours in the determined structure, so the conformation is not likely to be a consequence of crystal packing.

### A kinase C-terminal helix extends to the bound ATP

**Figure 2:**
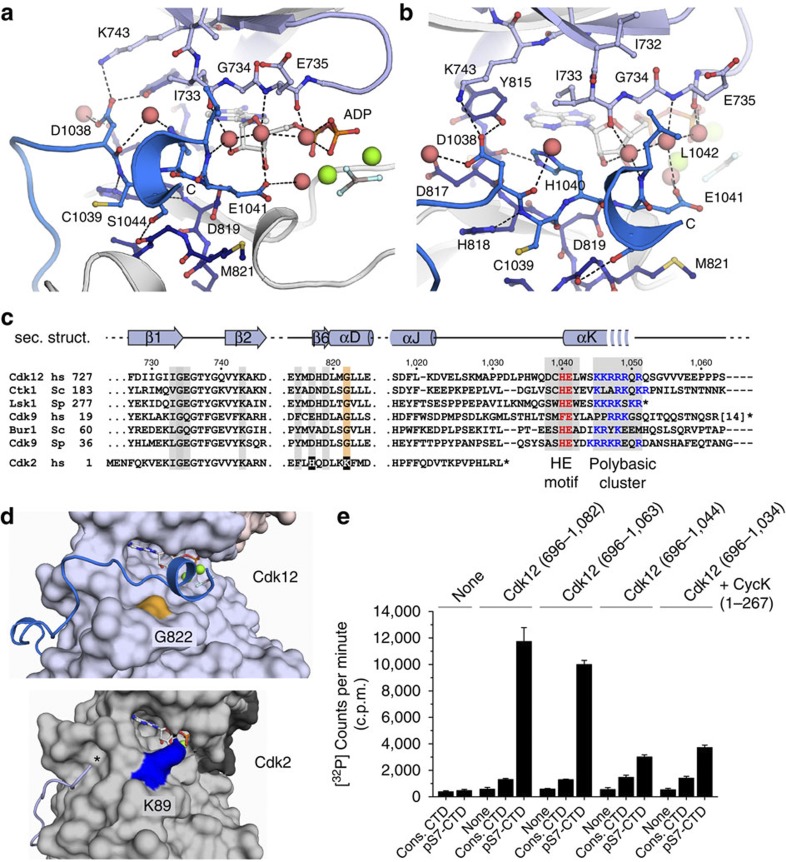
The C-terminal helix associates with the Cdk12 kinase core domain. (**a**) Close-up of DCHEL sequence interactions with the kinase core domain and the bound nucleotide. A network of weak water-mediated contacts is formed between E1041 and L1042 of the C-terminal helix and the hydroxyl groups of the ATP ribose that is stabilized by I733, E735 and T737 of the glycine-rich loop. (**b**) D1038 interacts with residues of the N- and C-terminal kinase lobe, while H1040 directly contacts the adenine base of the bound nucleotide. (**c**) Sequence alignment of the transcription-associated kinases Cdk12 and Cdk9 from human and their respective orthologous from *S. cerevisiae* and *S. pombe* compared with Cdk2. A C-terminal HE-motif followed by a polybasic region is conserved in the CTD kinases. Residues of the N- and C-terminal lobe that interact with the C-terminal helix are shaded grey. (**d**) Surface representations of Cdk12 (this study) and Cdk2 (3QHR; ref. 32) show the different accessibilities of the ATP ribose in the transcription-associated kinase and the cell cycle kinase. While a lysine residues (K89) in Cdk2 points towards the hydroxyl groups of the ATP ribose, this position is engaged with a small glycine in CTD kinases. (**e**) The activity of Cdk12 decreases upon truncation of the C-terminal helix. Four different length versions of Cdk12 were tested for their activity with two different kinase substrates, consensus CTD and pS7-CTD, using the same CycK (1–267) construct. Truncation of the polybasic cluster in Cdk12 from residue 1,063 to 1,044 led to a significant loss in activity for the pS7-CTD substrate. Data are the mean±s.d. of three independent experiments.

While crystallization attempts using a protein construct comprising only the canonical kinase domain were not successful, the structure showed that the protein extends by about 30 residues following the last helix αJ of the kinase domain fold (Supplementary Fig. 8). A stretch of 23 residues (1,017–1,039) meanders around the C-terminal kinase lobe to associate in the ATP cleft with the bound nucleotide. A ^1038^DCHEL motif reaches out to the N-terminal lobe, the ATP ribose and the C-terminal lobe, and interacts with the bound nucleotide by multiple water-mediated contacts (Fig. 2a,b). D1038 forms a salt bridge with K743, a polar interaction with Y815 and a water-mediated interaction with D817. The H1040 Nε2 amine interacts directly with the N3 nitrogen of the adenine base at 3.0 Å. The water-mediated interactions of E1041 and L1042 with the nucleotide are weaker, with distances of 2.3 and 3.9 Å, respectively. The HEL motif initiates a C-terminal helix that leads into the polybasic cluster ^1045^KKRRRQR, which is only partially resolved in the crystal structure (Fig. 2c and Supplementary Fig. 8b). This helix bundles in parallel orientation to the C-terminal lobe helix αD. The structure thereby resembles a ‘closed conformation’ for the bound nucleotide in which the free exchange of ADP for ATP is impaired by the intra-molecular contacts of the C-terminal helix formed with the kinase core domain.

The approach of the C-terminal helix αK towards the kinase active site is enabled by the presence of a small glycine (G822) in helix αD, which allows for association of this helix with the bound nucleotide (Fig. 2d). In Cdk2, this position is filled by a lysine (K89) whose amino group points towards the ATP ribose hydroxyl groups^32^. Interestingly, a glycine at this position is found in the human CTD kinases Cdk9, Cdk12 and Cdk13 as well as the *S. cerevisiae* and *S. pombe* orthologues Bur1, Ctk1, Cdk9 and Lsk1 (Fig. 2c). In contrast, human Cdk2, -3, -4, -5, -6, -7, -8 and -10 all contain bulkier residues such as lysine, threonine, valine, histidine or serine at this position, which might prevent association of a C-terminal extension with the nucleotide. The appearance of a glycine in helix αD thus correlates with the presence of the HE motif in the C-terminal extension that is followed by a polybasic cluster in the CTD kinases (Fig. 2c). Strikingly, progressive truncation of the C-terminal extension of Cdk12 led to a corresponding decrease in kinase activity. Whereas truncations from 1,082 to 1,063 produced only a small decrease in activity, truncation of the polybasic cluster at position 1,044 reduced its catalytic activity by approximately fourfold (Fig. 2e). However, additional shortening of the HE motif did not further reduce the kinase activity. These data suggest that the C-terminal extension of Cdk12 including its polybasic cluster is an important element for the catalytic activity of the kinase.

### Activation of Cdk12/CycK by CAK1

In contrast to Cdk9/CycT1 (refs 25, 34, 35), expression of human Cdk12/CycK in the baculovirus expression system did not result in T-loop phosphorylation of the kinase as determined by mass spectrometry. Although Cdk12/CycK kinase complex lacking T-loop phosphorylation showed some basal activity towards a CTD substrate prephosphorylated at position Ser7, its activity was significantly increased upon coexpression with the CAK from *S. cerevisiae* (Supplementary Fig. 9a). Mutation of T893 to E to mimic phosphorylation showed no effect on basal kinase activity. Quantitative phosphorylation of a single residue occurred upon coexpression with Cak1, as determined by ESI mass spectrometry (Supplementary Fig. 9b). The site of phosphorylation was identified as the activating T-loop residue T893 by peptide mass finger print analysis and confirmed in the crystal structure.

### Cdk12 prefers a prephosphorylated CTD substrate

**Figure 3:**
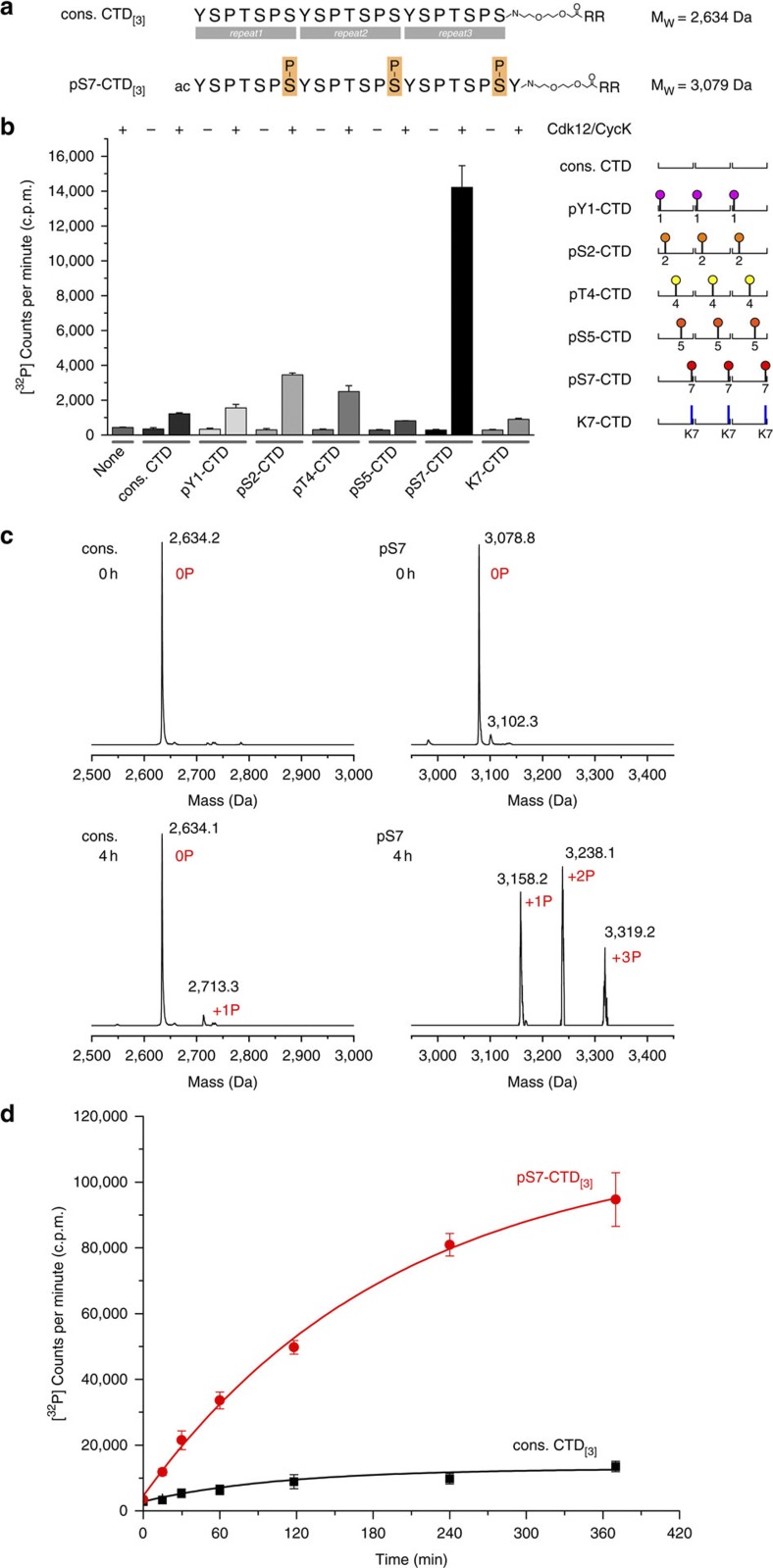
Cdk12 activity on the CTD requires Ser7 prephosphorylation. (**a**) Design of CTD substrate peptides used for kinetic and ESI-MS analyses. Peptides contained three consensus hepta-repeats with either no modification (cons. CTD_[3]_) or with phosphorylation marks continuously set at one residue of the heptad sequence as shown for Ser7 (pS7-CTD_[3]_). The same design principle was used for CTD peptides phosphorylated at Tyr1 (pY1-CTD_[3]_), Ser2 (pS2-CTD_[3]_), Thr4 (pT4-CTD_[3]_) and Ser5 (pS5-CTD_[3]_) or a peptide that contained a lysine at position 7 (K7-CTD_[3]_). (**b**) Activity of Cdk12/CycK for various CTD substrates. Of the seven peptides tested, Cdk12 showed the highest activity for continuous Ser7 prephosphorylation. Minor activities were detected for Ser2 and Thr4 prephosphorylated peptides. Remarkably, no activity was seen for cons. CTD_[3]_ or the K7-peptide, which is in contrast to P-TEFb. In addition, Cdk12 exhibited no activity on the pSer5 peptide. (**c**) ESI-MS analyses of CTD peptides before and after 4 h of incubation with 2 μM Cdk12/CycK. Whereas no phosphorylation occurred for the consensus peptide, the pS7-CTD_[3]_ substrate got readily phosphorylated up to three times. (**d**) Time course of Cdk12/CycK mediated CTD phosphorylation. The prephosphorylated Ser7 peptide got phosphorylated over time to saturation, while the non-phosphorylated peptide showed low susceptibility as a Cdk12 substrate. Data in panels **b** and **d** are the mean±s.d. of three independent experiments.

The highly variable patterns of phosphorylation marks in the hepta-repeats of Rpb1 are thought to regulate the elongation rate, RNA processing and chromatin modification^36^. Particular combinations of modifications may generate specific docking sites to recruit specific regulatory factors. We designed a series of synthetic CTD peptides, each containing three consensus hepta-repeats that were either unmodified or uniformly phosphorylated at all three Tyr1, Ser2, Thr4, Ser5 or Ser7 positions (Fig. 3a and Supplementary Table 1). The series also included a peptide containing substitution of serine to lysine at position 7. Surprisingly, despite its phosphorylation at the activating threonine T893 in the T-loop sequence, Cdk12/CycK showed low activity towards the unmodified consensus CTD peptide (Fig. 3b). This differs significantly from P-TEFb, which was highly active on the unmodified CTD, and could phosphorylate every hepta-repeat within an eight-repeat peptide in a 4-h incubation^34^. Among the prephosphorylated substrate peptides, pS7-CTD_[3]_ stood out as the optimal Cdk12 substrate. CTD peptides pTyr1, pSer2 and pThr4 were all phosphorylated more efficiently than the unmodified sequence, but even the best of these, pSer2, was modified at only 25% efficiency compared with pSer7. There was no activity above background towards the pSer5 peptide. Similarly, the YSPTSPK sequence was not phosphorylated by Cdk12. Again, this differs markedly from results with P-TEFb, which phosphorylates both consensus and K7-substituted CTD substrates to the same extent and with similar catalytic efficiency^34^. Analysis by mass spectrometry revealed that up to three phosphorylations were placed on the pS7-CTD substrate after 4-h incubation, while hardly any phosphorylation was detected on the unmodified peptide (Fig. 3c). This result was confirmed in time course experiments that showed the kinetics and saturation of the reaction (Fig. 3d). We conclude that Cdk12 prefers a substrate prephosphorylated at Ser7 to exhibit maximal kinase activity towards the CTD.

### Substrate preferences of Cdk12 and Cdk9 kinases

**Figure 4:**
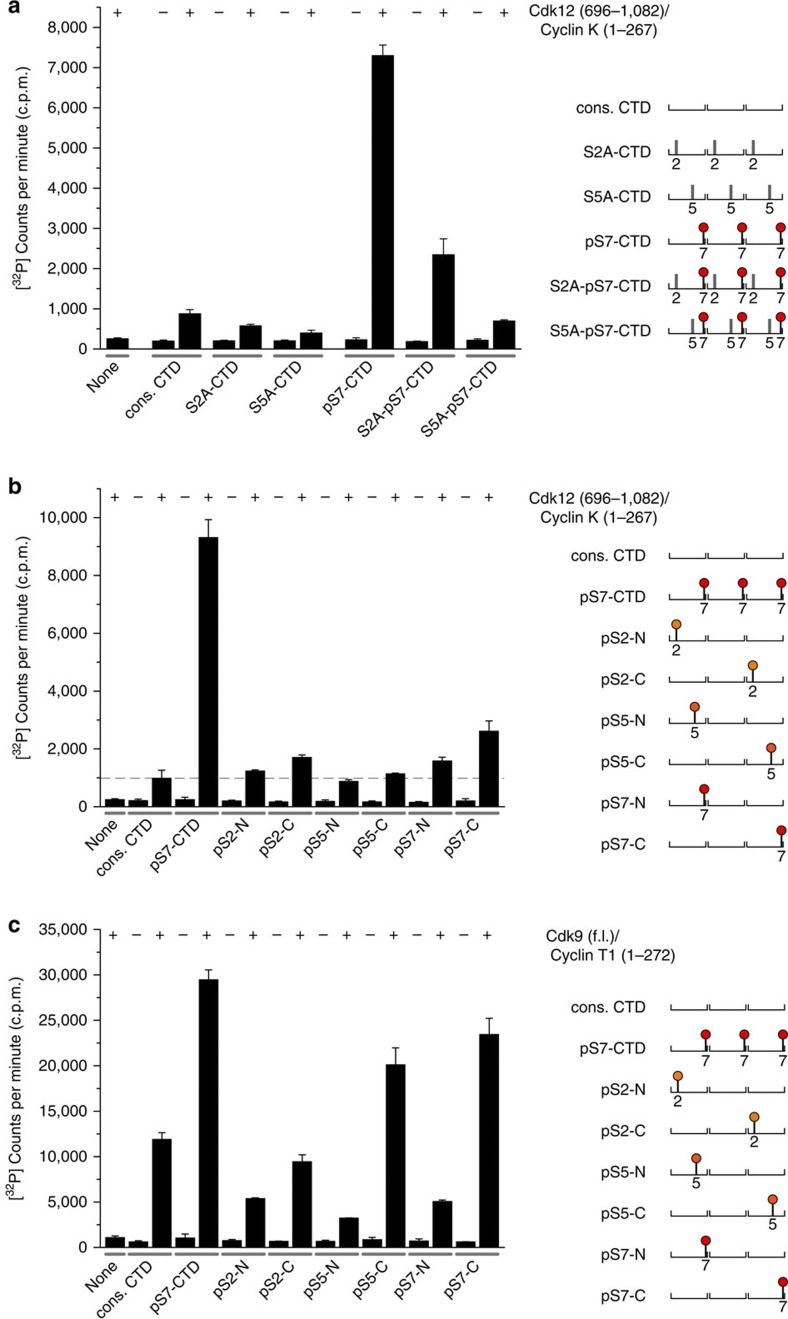
Substrate preferences of Cdk12 phosphorylation. (**a**) Activity of Cdk12 on CTD peptide substrates with serine-to-alanine mutations either at position 2 or at position 5 in combination with Ser7 pre-phosphorylations. Only minor activity was displayed on the three non-phosphorylated peptides cons. CTD, S2A-CTD and S5A-CTD. The pS7-CTD substrate instead was readily phosphorylated followed by the S2A-pS7-CTD peptide. The S5A-pS7-CTD peptide exhibited almost no susceptibility as a kinase substrate, suggesting that Cdk12 phosphorylates Ser5 in the context of Ser7 prephosphorylation. Cartoons displaying the peptide templates are shown right. (**b**) Priming and directionality of Cdk12 phosphorylation. Serine 2, 5 and 7 phosphorylation marks were set either at the N terminus or at the C terminus of triple hepta-repeats. While the activity of Cdk12 towards these peptides was overall very weak, the substrate with the pS7 mark set at the C terminus gained highest recognition. (**c**) In contrast, Cdk9 exhibits high activity towards C-terminally phosphorylated peptides at either position 5 or position 7, suggesting the priming of the kinase by these prephosphorylations. All data are reported as the mean±s.d. from three independent experiments.

To explore Cdk12-mediated CTD phosphorylations in more detail, we performed mutagenesis experiments and western blot analyses with either synthetic CTD peptides or native full-length RNA Pol II CTD. Using a similar strategy as described above, four CTD peptides were generated that contained alanine at either position 2 or position 5 within each repeat, both with and without Ser7 prephosphorylations in each repeat (Fig. 4a). As before, there was little activity towards the non-phosphorylated substrates. However, when the Ser7 position was phosphorylated, the S2A peptide was a better substrate than the S5A peptide, indicating that Cdk12 can phosphorylate Ser5. In a second set of experiments, we placed single Ser2, Ser5 or Ser7 phosphorylation marks at either end of the three-repeat substrate (Fig. 4b). Activity of Cdk12/CycK towards any of these peptides only marginally exceeded that towards the unphosphorylated peptide, with the peptide containing a single pS7 mark at the C terminus being the best substrate. However, the phosphorylation activity was less than one-third of that towards the uniformly phosphorylated pS7-CTD_[3]_ substrate. In contrast, when we used P-TEFb to phosphorylate the same peptides, both pS5-C and pS7-C, but not their N-terminal counterparts, were phosphorylated to a greater extent than the unphosphorylated peptide (Fig. 4c), in line with the recently described priming of the CTD by previous phosphorylation marks^18^,^24^,^34^. These data suggest that Ser7 prephosphorylations cause no priming effect of the CTD for Cdk12 activity but are instead necessary elements of the kinase recognition motif.

### Cdk12/CycK is a promiscuous CTD kinase

**Figure 5:**
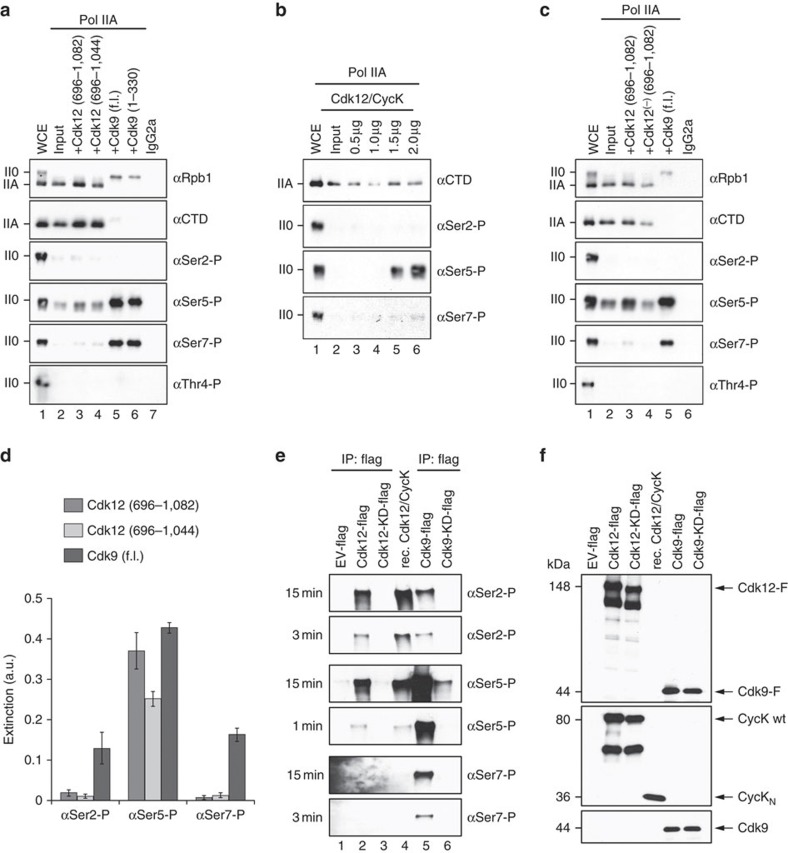
Cdk12/CycK is a promiscuous CTD kinase. Immunoprecipitated Pol IIA with the antibody 1C7 from HeLa whole-cell extract was used as substrate for Cdk12 and Cdk9, respectively, and subjected to western blot analysis using mAbs specific for Rpb1 (Pol 3.3. and 1C7, the latter recognizes non-modified CTD) or CTD phosphorylations pSer2 (3E10), pSer5 (3E8), pSer7 (4E12) and pThr4 (6D7). IgG2a is an isotype control. The hyper- (II0) and hypophosphorylated forms (IIA) of Pol II are indicated. The Pol IIA form corresponds to an aberrant molecular mass of 250 kDa. (**a**) Comparison of Cdk12 and Cdk9 CTD substrate specificities. Incubation of Rpb1 with Cdk12 results in phosphorylation of Ser5 and to a small extent Ser7, which decreased upon C-terminal truncation of the kinase. Cdk9 showed similar substrate specificity albeit at a significantly higher enzymatic activity. (**b**) Cdk12 (696–1,082) phosphorylates Pol IIA at Ser5 residues, and to a lesser extent at Ser7 residues, in a concentration-dependent manner. (**c**) T-loop phosphorylation enhances the activity of Cdk12/CycK for Ser5 phosphorylation. Incubation of the RNAPII substrate with phosphorylated Cdk12 (lane 3), non-phosphorylated Cdk12 (lane 4) or Cdk9 (lane 5) showed a significantly enhanced Ser5 phosphorylation for the activated Cdk12 kinase. (**d**) Quantitative ELISA data for Cdk12 and Cdk9 kinase activity on a CTD peptide. A CTD consensus peptide of two hepta-repeats was used as kinase substrate. Error bars show s.d. of three independent experiments. (**e**) *In vitro* kinase assays with Cdk12 and Cdk9 and GST-tagged full-length human CTD as a substrate. Flag-tagged kinases were immunoprecipitated from HCT116 cells and compared with recombinant Cdk12 (696–1,082)/CycK (1–267). Recombinant and flag-tagged Cdk12 phosphorylate Ser2 and Ser5 to the same extent (lanes 2 and 4). Flag-tagged Cdk9 strongly phosphorylates Ser5 and to lesser extent Ser7 and Ser2 (lane 5). EV indicates the empty vector control. Exposure times are indicated on the left. (**f**) Display of the protein input for the *in vitro* kinase assays by western blot analysis. Equal amounts of proteins were used in *in vitro* kinase assays as shown by probing western blots with anti-flag (upper panel) and anti-CycK (middle panel) antibodies. Cdk12 purifications are free of Cdk9 contaminants as shown by western blot analysis with an anti-Cdk9 antibody (lower panel).

To further compare Cdk12 and Cdk9 kinase specificities, we performed western blot analyses using anti-phospho-CTD-specific antibodies raised against pSer2, pSer5, pSer7 and pThr4 phosphorylation marks. The hypophosphorylated form of RNA Pol II, Pol IIA, was purified from HeLa cell extracts using a specific α-Rpb1 antibody, as described^37^. The substrate was incubated with recombinant Cdk12/CycK or Cdk9/CycT1 for 60 min (Fig. 5a). In addition to comparing Cdk12 to Cdk9, we also compared two different variants of each kinase, which differed in the length of the C-terminal extension appended to the catalytic domain. Whereas Cdk9 showed a strong preference for Ser5 phosphorylation followed by Ser7 but not Ser2, Cdk12 showed a preference for Ser5 but could also catalyse Ser7 phosphorylation, albeit with much lower catalytic activity than Cdk9. Truncation of the kinase length from residue 1082 to 1044 in Cdk12 led to a reduction in activity. No activity was seen towards Thr4, however, for any kinase. A titration from 0.5 to 2.0 μM Cdk12/CycK showed increasing phosphorylations of Ser5 with a faint band observed for Ser7 phosphorylation at high concentrations (Fig. 5b). The Cdk12 kinase activity depended on T-loop phosphorylation, as incubation of the RNA Pol II substrate with phosphorylated Cdk12 (lane 3), non-phosphorylated Cdk12 (lane 4) or Cdk9 (lane 5) showed a significantly enhanced Ser5 phosphorylation by the activated kinase (Fig. 5c). Quantitative ELISA data for Cdk12 and Cdk9 kinase activity using a tandem-repeat consensus CTD peptide revealed highest efficacy towards Ser5 phosphorylation for Cdk12 that decreased upon truncation of the kinase C-terminal HE-motif and polybasic region (Fig. 5d). Similarly, Cdk9 showed Ser5 phosphorylation activity but also phosphorylation of Ser7 and Ser2 after 60-min incubation. The latter results were confirmed by time course experiments of P-TEFb that underlined the restriction in the recognition specificity of monoclonal antibodies against multiply- phosphorylated CTD epitopes (Supplementary Fig. 10).

To compare the results of recombinant Cdk12 kinase domains with native full-length protein, we first expressed and purified human full-length Cdk12 as a GST fusion protein in baculo-infected insect cells using the same strategy with CycK and Cak1 coexpression as before. The protein complex was expressed and purified in triplicate, and the identity of Cdk12 confirmed by peptide mass finger print analysis (Supplementary Fig. 11a). In the kinase assay with immunoprecipitated Rpb1, no significant increase in CTD phosphorylation could be observed suggesting the lack of kinase activation signals (Supplementary Fig. 11b). We therefore overexpressed flag-tagged full-length Cdk12 and Cdk9 as well as the kinase dead variants Cdk12 D877N and Cdk9 D167N in HCT116 cells, respectively, and compared their activity with recombinant Cdk12/CycK. Incubation of GST-CTD with anti-flag immunoprecipitated Cdk12 in an *in vitro* kinase assays revealed the strongest signal for Ser5 phosphorylation closely followed by Ser2 phosphorylation, while no pSer7 mark was seen (Fig. 5e). The empty vector control and the kinase dead variants showed no phosphorylations of the CTD. Importantly, the recombinant Cdk12 (696–1,082)/CycK (1–267) protein complex exhibited the same phosphorylation specificity as the flag-tagged native complex. Cdk9/CycT1 showed by far the highest activity towards Ser5 phosphorylation followed by pSer7 and pSer2 marks. Input controls of Cdk12/CycK and Cdk9/CycT1 by western blot analyses confirmed the integrity of Cdk–cyclin complexes and equal amounts of kinases in the *in vitro* kinase assays (Fig. 5f). Together, these data suggest Cdk12 is a promiscuous CTD kinase that has a preference for Ser5 phosphorylations in the *in vitro* kinase assays.

### Inhibition of Cdk12 compared with Cdk9

**Figure 6:**
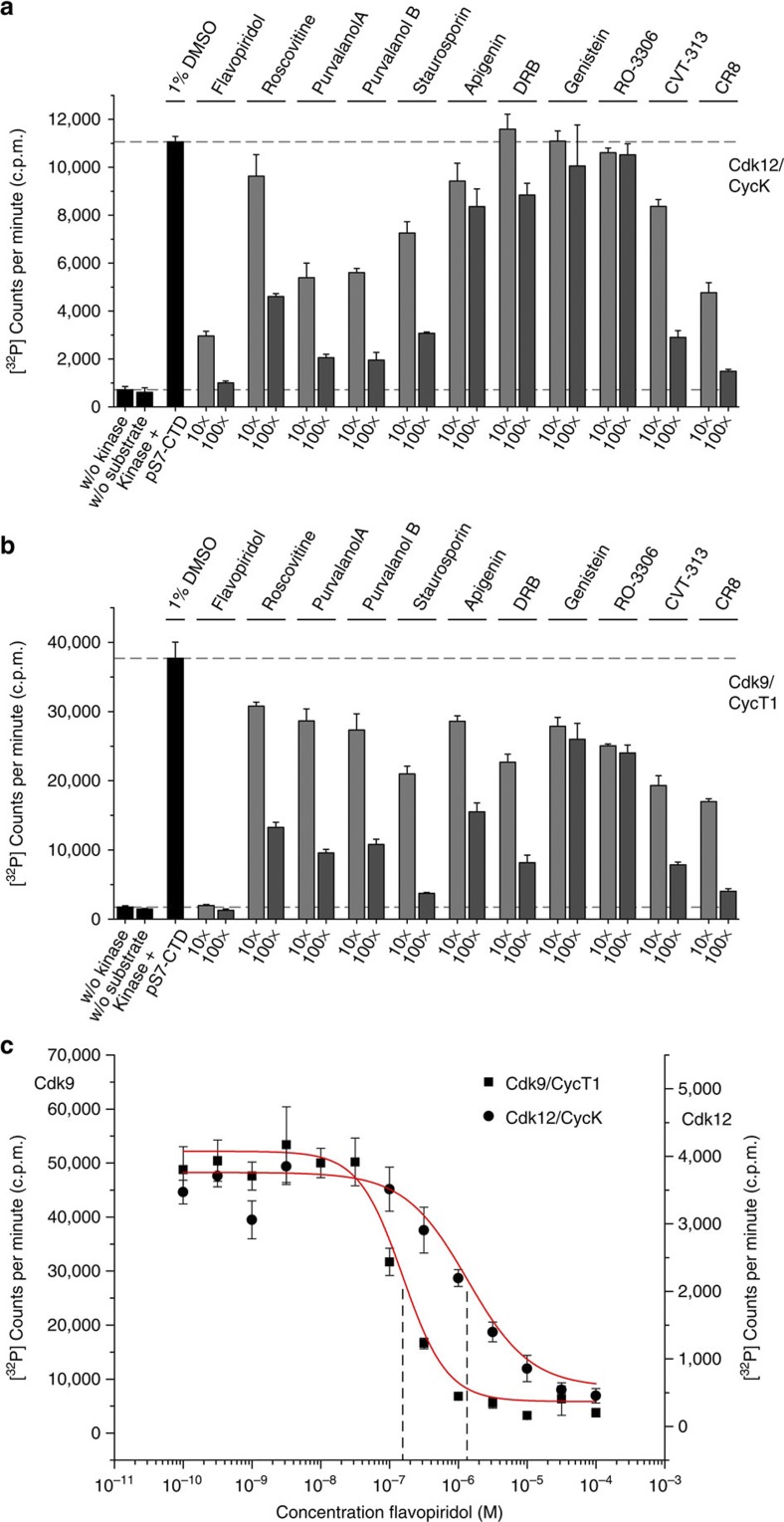
Inhibition of CTD kinases Cdk12 and Cdk9 by small molecule inhibitors. (**a**) A panel of eleven small molecule inhibitors was tested at 10- and 100-fold molar excess against the kinase activity of Cdk12/CycK. Flavopiridol showed the highest efficacy for Cdk12 inhibition followed by CR8. (**b**) The same panel of inhibitors was analysed for Cdk9/CycT1 inhibition. Strongest inhibition was indeed achieved with flavopiridol, whose potency to inhibit Cdk9 turned out to be at least one order of magnitude higher than the closest followers, CR8 and Staurosporin. (**c**) Concentration series of flavopiridol for Cdk12/CycK and Cdk9/CycT1 at 0.2 μM kinase concentration. The IC_50_ values were determined to be 1.37 μM against Cdk12 and 0.144 μM against Cdk9 using pS7-CTD_3_ as substrate. All data were reported as the mean±s.d. from three independent experiments.

We tested a series of kinase inhibitors for their ability to inhibit Cdk12/CycK or P-TEFb. To a concentration of 0.5 μM Cdk12/CycK a 10- or 100-fold excess of inhibitors was added, using 1 mM ATP and 100 μM pS7-CTD_[3]_ substrates. Whereas some compounds such as DRB, Genistein, RO-3306 and Apigenin showed almost no inhibitory effect on Cdk12, Purvalanol A and B, and CR8 reduced Cdk12 activity nearly 10-fold at 100-fold excess concentration (Fig. 6a). Roscovitine, Staurosporine and CVT-313 showed more modest activity towards Cdk12. The highest efficacy, however, was achieved with flavopiridol. Flavopiridol is known as a potent ATP competitive kinase inhibitor for Cdk9 that is now in clinical trials^25^,^38^.

In contrast, inhibition of P-TEFb was more potent for all of the inhibitors tested (Fig. 6b). Roscovitine, Purvalanol A and B, Apigenin, DRB and CVT-313 were all of intermediate potency towards P-TEFb, whereas only Genistein and RO-3306 were found to be weak inhibitors. Staurosporine and CR8 were of higher potency against Cdk9, but were still >10-fold less potent than flavopiridol, which inhibited P-TEFb activity by ~20-fold even at the lower dose. Because flavopiridol was found to be the best inhibitor against both Cdk9 and Cdk12, we determined comparative *in vitro* IC_50_ values at 0.2 μM kinase concentration (Fig. 6c). Although the potency of flavopiridol towards Cdk12/CycK was about one order of magnitude weaker than that for Cdk9, the data suggest that flavopiridol is a specific inhibitor of the CDKs active in transcriptional elongation.

## Discussion

The structure of Cdk12/CycK contains a C-terminal extension outside the canonical kinase lobes that appears unique for CTD kinases implicated in transcription elongation and whose truncation correlates with a loss of activity. Conformational transitions are essential elements of the regulation and reaction mechanism of protein kinases, and the closing together of the N- and C-terminal lobes is a characteristic structural feature of protein kinase activation^39^,^40^. Several kinases have been reported in the past to contain C-terminal extensions that are responsible for their regulation. The calcium/calmodulin-dependent protein kinase I, for example, contains a C-terminal helix-loop-helix segment that interacts with both kinase lobes and keeps the catalytic centre in an autoinhibited state^41^. For Cdk12, the function of the HE motif when contacting the ATP substrate could be similar to protein kinase A, whose C-terminal tail undergoes a rearrangement to stabilize the kinase conformation during the catalytic cycle^31^,^33^. The kinetic mechanism of substrate binding and product release, however, is complicated by the multiple phosphorylation sites in a single chain of the CTD. A crystal structure containing the C-terminal tail sequence of Cdk9 was recently reported, suggesting an ordered mechanism in which the phosphorylated protein is the first product to leave the enzyme active site^42^. The contribution of the polybasic motif identified here to the enzymatic activity of Cdk12 supports such mechanism for the association of the negatively charged CTD substrate.

The substrate specificity of Cdk12/CycK for CTD serine phosphorylations was analysed in different kinase assays, using either the recombinant kinase domain in combination with the cyclin box domain of CycK purified to homogeneity, full-length recombinant Cdk12 or immunoprecipitated full-length Cdk12 expressed from human cells. Similarly, multiple substrates were used such as synthetic CTD peptides up to a length of three hepta-repeats that were modified with prephosphorylation signatures or site-specific alanine mutations, a full-length GST-CTD construct containing all 52 human repeats expressed from *E. coli* or a precipitated form of human Rpb1 from HeLa cells with the CTD in the hypophosphorylated IIA state. All assays show in common that Cdk12 predominantly phosphorylates Ser5 of the CTD, determined either by mutagenesis experiments or by western blot analysis. However, while the use of Rpb1 showed a faint band for Ser7 phosphorylation but none for Ser2 phosphorylation, flag-tagged full-length Cdk12 exhibited also Ser2 phosphorylation in the GST-CTD substrate but not Ser7 phosphorylation. The full-length recombinant Cdk12 kinase expressed from insect cells instead showed no kinase activity, indicating either the loss of activation signals or autoregulatory motifs in regions adjacent to the kinase domain. Together, these data suggest that Cdk12 is a promiscuous CTD kinase that has a preference for Ser5 phosphorylations. It should, however, be noted that co-factors of Cdk12/CycK in cells could change its catalytic activity or even modulate its substrate phosphorylation profile.

**Figure 7:**
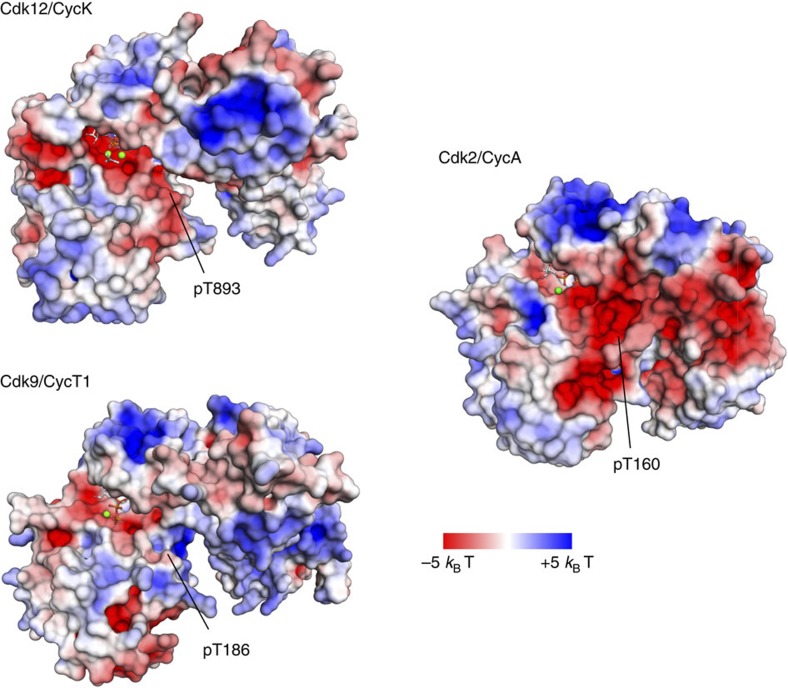
Electrostatic surface display of transcription and cell cycle associated Cdk/cyclin pairs. Basic patches at the surface of Cdk12/CycK (4NST, this study) and Cdk9/CycT1 (3BLQ,ref. 25) surrounding the active site of the kinase may accomplish the recognition of the highly negatively charged RNA Pol II CTD. In contrast, the cell cycle regulating Cdk2/CycA (1JST, ref. 58) heterodimer is largely negatively charged, in line with the recognition specificity for an SPx(K/R) motif for the kinase domain and an RxL motif at the cyclin domain. Note that the polybasic cluster at the C terminus of the Cdk12 kinase domain is not contained in this display. Electrostatic surface charge is shown from −5 *k*_B_T (red) to +5 *k*_B_T (blue) for all three Cdk/cyclin complexes.

Experiments using either recombinant or immunoprecipitated P-TEFb confirm Cdk9 as a Ser5 CTD kinase as recently described^34^. A similar observation has been made by *in vivo* live imaging experiments of RNA Pol II transcription factories in primary cells, where Cdk9 foci colocalized with pSer5 but not pSer2 marks^43^. Chromatin immunoprecipitation assays coupled with deep sequencing (ChIP-seq) showed that the genome-wide occupancy of Cdk9 is similar to RNA Pol II Ser5 phosphorylation, with the highest enrichment in the 5′-end of genes^43^. The various substrate specificities are partly contained in the electrostatic surface characteristics of the Cdk–cyclin complexes that allow association with the negatively charged CTD substrate when phosphorylated (or primed) at neighbouring repeats. Different basic surface patches are indeed seen for the catalytic sites of Cdk12/CycK compared with Cdk9/CycT1 that could account for the kinase phosphorylation activities (Fig. 7). The cell cycle regulating Cdk2/CycA complex instead exhibits a largely acidic surface at the kinase active site, underlining its preference for the SPx(K/R) and RxL substrate recognition motifs^26^.

It is surprising that in the *in vitro* kinase assays Cdk12 exhibits low activity towards a CTD that is not prephosphorylated at Ser7. This could indicate an additional layer of regulation as Cdk12 is only poorly able to phosphorylate a CTD in the preinitiation state but rather requires other kinases to place phosphorylation marks before it properly recognizes the CTD as a substrate. There are three main differences in the activity and substrate specificity of Cdk12 compared with those of Cdk9: (i) Cdk12 requires a preset pSer7 mark for full activity on the CTD, whereas Cdk9 readily phosphorylates the consensus CTD as well as the pSer7 prephosphorylated CTD; (ii) Cdk12 does not phosphorylate a repeat containing Lys7, whereas Cdk9 does; and (iii) Cdk12 appears to place only one phosphorylation mark per pSer7 mark, whereas Cdk9 is able to phosphorylate a CTD substrate at a stoichiometry equalling the number of CTD repeats provided. A preset pSer7 or pSer5 mark particularly in a C-terminal repeat of a CTD substrate stimulates Cdk9 activity, whereas Cdk12 seems to be able to place only one phosphorylation N terminally to the pSer7 mark. The state of premodifications in CTD substrates could be indeed another source of ambiguity between *in vitro* and *in vivo* analyses that is, however, inherently linked to the periodic hepta-repeat structure of the substrate.

The misregulation of transcriptional regulation is increasingly recognized as a cause of a broad range of diseases^44^ and changes in the *Cdk12* gene, and its expression might predispose to human disease. Mutations of *Cdk12* have been identified in genomic analyses of ovarian, breast and lung carcinoma and melanoma^45^,^46^,^47^. Several of these mutations align with the kinase domain of the 164 kDa protein, suggesting the catalytic activity and interaction with CycK are crucial for cell viability. The structure of human Cdk12 with its C-terminal helix associating with the ATP substrate creates the basis for site-directed development of specific inhibitors, for example, the dynamics of the C-terminal extension could be confined by displacing water molecules from this unique interface to the ATP cosubstrate.

The decipherment of the CTD code requires the understanding of the setting of modifications, their recognition and their removal in a spatial and temporal manner, to link these transient modifications to the transcription cycle of various genes^48^,^49^. The determination of different substrate specificities and enzymatic activities in the two mammalian kinases Cdk9 and Cdk12 is a first step to recognize their contributions to the variability in CTD phosphorylations. The identification of Cdk12/CycK regulating factors will be a next step to understand its function in the regulation of transcriptional elongation.

## Methods

### Plasmids and proteins

Expression and purification of Cdk12/CycK protein complexes were carried out in insect cells using the MultiBac^Turbo^ system^50^. Synthetic genes comprising the kinase domain of human Cdk12 (UniProt accession number Q9NYV4; residues 696–1,082) and the cyclin box domain of human CycK (O75909; residues 1–300) codon optimized for expression in *Trichoplusia ni* were synthesized by GeneArt (Regensburg). Cdk12 was cloned into a modified pACEBac1 acceptor vector (ATG:biosynthetics) including an N-terminal GST affinity tag followed by a TEV (tobacco etch virus protease) cleavage site. CycK was similarly cloned with a TEV-cleavable N-terminal GST affinity tag into the pIDK donor vector. Full-length human Cdk12 was cloned from a cDNA clone (GenBank accession number BC150265.1) purchased from imaGenes (Source BioScience) with restriction sites EcoR1 and Not1 and inserted with an N-terminal GST tag followed by the TEV site into the pACEBac1 expression vector. Full-length CAK1 (P43568) from *S. cerevisiae* was cloned into the pIDC donor vector without any affinity tag. All plasmids were confirmed by DNA sequencing before expression.

Vectors were fused by *in vitro* Cre recombination and applied to Tn7-dependent integration into the baculoviral genome of DH10 MultiBac^Turbo^ cells (ATG:biosynthetics). Recombinant bacmid DNA was isolated and then used to transfect *Sf21* insect cells. Liquid culture of *Sf21* cells was maintained at 27 °C in SF-900 III SFM medium (Invitrogen) shaking at 100 r.p.m. Initial recombinant baculoviruses were amplified in *Sf21* cells and used for expression by infecting cells at a density of 1.5 × 10^6^ cells per ml by addition of 2% (v/v) of virus stock V2 (multiplicity of infection (MOI)>1). After 72–96 h, cells were collected by centrifugation, washed in PBS and pellets stored at −80 °C.

For large-scale purification of CDK12/CycK constructs, cells were resuspended in lysis buffer (50 mM Hepes pH 7.6, 500 mM NaCl, 10% glycerol and 1 mM DTT) and disrupted by sonication. The lysate was cleared by centrifugation in a Beckman Optima L-80 XP Ultracentrifuge with a Ti45 rotor (45,000 r.p.m. for 45 min at 4 °C) and applied to GST Trap FF columns (GE Healthcare) equilibrated with Lysis buffer using an Äkta Prime chromatography system (GE Healthcare). Following extensive washes with 10 column volumes (CV) of Lysis buffer and 5 CV of wash buffer (50 mM Hepes pH 7.4–8.2, 1,000 mM NaCl, 10% glycerol and 1 mM DTT), the protein was eluted in elution buffer (50 mM Hepes pH 7.4–8.2, 500 mM NaCl, 10% glycerol and 1 mM DTT, 10 mM Glutathione).

Cleavage of the GST tag was achieved by adding TEV protease in a 1/20 ratio and was performed for 20 h at 4 °C. Protein solution was concentrated and loaded on a preparative HiLoad 16/60 Superdex 200 prep grade gel filtration column (GE Healthcare) equilibrated in GF buffer (20 mM Hepes pH 7.4–8.2, 400 mM NaCl, 5% glycerol and 2 mM TCEP). Fractions of the main peak containing pure Cdk12/CycK complex as determined by SDS–PAGE were pooled and concentrated. The protein was aliquoted, snap frozen in liquid nitrogen and stored at −80 °C. Cdk9/CycT1 as well as human full-length GST-CTD proteins were prepared similarly as described^34^.

### Crystallization and structure determination

For crystallization, the purified Cdk12/CycK complex was mixed at 85 μM concentration with ADP, AlF_3_, MgCl_2_ and substrate peptide P-pS-YSPTSP-pS-YSPT in molar ratios of 1:8:32:64:8 and incubated on ice for 30 min. Initial crystals were obtained using the hanging drop vapour diffusion technique at 293 K by mixing 1 μl protein solution with 1 μl of the reservoir solution containing 0.1 M Bis-Tris, pH 6.5, 25% PEG 3350 and 0.325 M MgCl_2_. Crystals grew as clusters that showed high mosaicity while testing on the diffractometer. Micro-seeding (‘Beads for Seeds’ from Jena Biosciences) was used to obtain large single crystals. The seed stock was obtained by transferring an entire drop with crystals to a microcentrifuge tube containing glass beads and 100 μl of stabilization solution (0.1 M Bis-Tris, pH 6.5, 20.5% PEG 3350 and 0.4 M MgCl_2_). The seed stock was vortexed for 2 min and serial dilutions were prepared.

Crystallization drops were set up by mixing protein sample and seed stock dilutions in a 1:1 ratio and crystals were grown using the hanging drop vapour diffusion technique at 293 K. Best crystals grew within 2 weeks to a size of about approximately 200 × 30 × 30 μm^3^ using a 10^−2^ dilution of the seed stock. For cryo-protection, crystals were transferred to a solution that contained the stabilizing agents with additional 0.4 mM substrate peptide and 15% ethylene glycol. After 5–10 s soaking, crystals were flash frozen in liquid nitrogen.

Diffraction data were collected at the Swiss Light Source Villigen at 0.9785 Å wavelength and 100 K temperature using the PILATUS 6M detector (oscillation width per frame: 0.25°; 720 and 740 frames collected). The XDS package^51^ was used to process, integrate and scale the data. The structure was solved by molecular replacement using the program PHASER^52^ and the coordinates of Cdk9 (3BLQ^25^) and CycK (2I53 (ref. 53)^53^) as search model. The model was refined by alternate cycles of refinement using REFMAC5 (ref. 54)^54^ and PHENIX^55^. The manual rebuilding was made using the graphical program COOT^56^. Protein interfaces and accessible surface areas were calculated with the program ePISA (http://www.ebi.ac.uk/pdbe/). Molecular diagrams were drawn using PyMOL (http://www.pymol.org/).

### RNA Pol II CTD substrate peptides

For kinase activity analyses, various CTD polypeptides were purchased from Biosyntan (Berlin) or synthesized in house with 95% purity (HPLC grade; see Supplementary Table 1). The peptide used for crystallization (ac-P-pS-YSPTSP-pS-YSPT-amid) contained two phosphorylated serine residues at heptad position 7. For quantitative analysis in ESI-MS experiments or radioactive filter-binding assays, CTD substrate peptides were marked at the C terminus with a double arginine motif separated by a polyethylene glycol spacer for better ionization properties or increased transfer rates, respectively.

### *In vitro* kinase assays

Radioactive kinase reactions (typically 35 μl) were carried out with recombinant highly purified proteins using a standard protocol similarly as described^34^. In short, Cdk12/CycK (0.5 μM) was preincubated with CTD substrates (peptides 100 μM; GST-CTD f.l. 2.5 μM) for 10 min at room temperature in kinase buffer (150 mM Hepes pH 7.6, 34 mM KCl, 7 mM MgCl_2_, 2.5 mM dithiothreitol, 5 mM β-glycerol phosphate, 1 × PhosSTOP (Roche)). Cold ATP (to a final concentration of 1 mM) and 3 μCi [^32^P]-γ-ATP were added, and the reaction mixture was incubated for 60 min at 30 °C at 500 r.p.m. Reactions were stopped by adding EDTA to a final concentration of 50 mM. For reactions using GST-CTD, aliquots of 11 μl each were spotted onto P81 Whatman paper squares. For substrate peptides Optitran BA-S85 reinforced membrane was used. Paper squares were washed three times for 5 min with 0.75% (v/v) phosphoric acid, with at least 5 ml washing solution per paper. Radioactivity was counted in a Beckman Scintillation Counter (Beckman-Coulter) for 1 min. Measurements were performed in triplicate and are represented as mean with s.d.

For ELISA, the consensus CTD-peptide (YSPTSPSYSPTSPSC; Peptide Specialty Laboratories GmbH, Heidelberg) was coupled to 96-well maleimide plates for 60 min at 37 °C in carbonate buffer at pH 9.5. After washing, the kinase assay was performed using recombinant Cdk12 (1.5 μg) or Cdk9 kinase (0.5 μg) in 25 μl kinase buffer (20 mM Tris/HCl (pH 7.4), 20 M NaCl, 10 mM MgCl_2_, 1 μM DTT, 1 × PhosSTOP and 2 μM ATP) at 28 °C for 60 min followed by washing and blocking with PBS/milk (1%) for 30 min. Primary antibodies were added and incubated for 30 min. After an additional washing and blocking step, biotin-coupled secondary antibodies were added for 30 min. Following another washing and blocking step, peroxidase attached to avidin was added to the wells. After washing five times with PBS, 50 μl of substrate buffer (o-phenylenediamine and H_2_O_2_; pH 5.0) was added. After colour change, OPs of samples were measured at 405 nm in the ELISA reader.

For the kinase assay with the endogenous Pol II as substrate, Pol II was immunepurified from whole HeLa cell extracts with an antibody recognizing Pol IIA with non-phosphorylated CTD (1C7). Five microlitres of the substrate-coupled Sepharose G beads was incubated with 45 μl kinase buffer B (50 mM Hepes (pH 7.9), 100 mM KCl, 10 mM MgCl_2_, 200 μM EGTA, 100 μM EDTA, 1 mM DTT, 200 μM ATP, 1 μg BSA, 1 × PhosSTOP and 1.5 μg of the recombinant Cdk12 kinase or 0.5 μg of recombinant Cdk9 kinase) at 30 °C for 60 min. Laemmli buffer was added (sixfold) and samples were incubated for 5 min at 95 °C followed by western blot analysis.

### Kinase assays with Flag-tagged proteins

Plasmids pcDNA3.1 3xFlag Cdk12 and pcDNA3.1 3xFlag Cdk9 used for expression of full-length flag-tagged proteins as well as the pcDNA3.1 3xFlag empty vector were described previously^16^. Kinase dead mutants Cdk12 D877N and Cdk9 D167N were obtained by mutagenesis of the corresponding wild-type plasmids and cloned into the pcDNA3.1 3xFlag expression vector.

One 15-cm plate of HCT116 cells was transfected with 20 μg of plasmid using PEI reagent. After 48 h, cells were collected and lysed in lysis buffer (20 mM Hepes/KOH pH 7.9, 15% glycerol, 0.2% NP-40, 300 mM KCl, 1 mM DTT, 0.2 mM EDTA and protease inhibitor (Sigma)). Flag-tagged proteins were immunoprecipitated from the lysate using 20 μl of flag-agarose (Sigma). The immunoprecipitates were washed three times with 1 ml of the lysis buffer containing 500 mM KCl followed by washing with 1 ml of a detergent-free buffer (20 mM Hepes/KOH pH 7.9, 150 mM KCl, 1 mM DTT, 15% glycerol). The flag-tagged proteins were eluted from the flag-agarose with 40 μl of flag peptide dissolved in 20 mM Hepes pH 7.9, 150 mM KCl, 1 mM DTT. *In vitro* kinase assays were performed in 20 mM Hepes/KOH pH 7.9, 5 mM MgCl_2_, 2 mM DTT and 1 mM ATP with either 10 μl of flag eluate or 40 ng of recombinant CycK/Cdk12 and with 300 ng of GST-tagged human full-length CTD as a substrate. Total of 60 μl of kinase reaction was incubated at 30 °C for 1 h, and reaction was terminated by adding 60 μl of 2 × SDS sample buffer.

### Immunoprecipitations

A total of 4 × 10^6^ HeLa cells were lysed in 200 μl IP buffer (50 mM Tris-HCl, pH 8.0, 150 mM NaCl, 1% NP-40 (Roche), 1 × PhosSTOP (Roche), 1 × protease inhibitor cocktail (Roche)) for 20 min on ice. All samples were sonicated on ice using a BRANSON Sonifier 250 (15 s on, 15 s off, 50% duty) and centrifuged at 14,500 r.p.m. for 15 min at 4 °C. The supernatant was incubated with 50 μl antibody-coupled protein G-sepharose beads (2.5 μg of antibody 1C7 for 4 h at 4 °C, followed by 2 washes with 1 ml IP buffer) rotating overnight. Beads were washed six times with 1 ml IP buffer and used as substrate for *in vitro* kinase assay.

### Western blots

Samples of protein were collected following treatment using 6 × Laemmli buffer. Protein was subjected to SDS–PAGE on a 6.5% gel before transfer to nitrocellulose (GE Healthcare). Membranes were stained with affinity-purified, IR-labelled secondary antibodies against rat (680 nm; Alexa, Invitrogen) and mouse (800 nm; Rockford, Biomol), and stained with hrp-conjugated secondary antibodies against rat (Sigma) or mouse (Promega), and revealed by enhanced chemiluminescence.

The monoclonal antibodies specific for Rpb1 (Pol3.3 and 1C7; the latter recognizes non-modified CTD) and the different CTD phosphorylations (3E10, 3E8, 4E12) were diluted in a 5% milk solution (0.1% Tween in PBS) at a 1:10 ratio. The rat monoclonal antibody 6D7 against CTD-Thr4-P was applied in a 2% ECL Advance blocking agent solution (GE Healthcare) (0.1% Triton X-100 in PBS) at a 1:10 dilution as described previously^37^,^57^.

### Kinase inhibition assays

Apigenin, Genistein, Staurosporine (from *Streptomyces sp*.), DRB (5,6-Dichlorobenzimidazole 1-β-D-ribofuranoside), Purvalanol A, Purvalanol B and CR8 were purchased from Sigma Aldrich. Roscovitine was purchased from Merck Chemicals, and RO-3306, CVT-313 and Flavopiridol were bought from Enzo Life Sciences. All compounds were dissolved in 100% DMSO to a stock solution at a concentration of 10 mM.

### Mass spectrometry analyses

Peptide and protein masses were determined by liquid chromatography-electrospray ionization-mass spectrometry using an Agilent 1100 chromatography system and an LCQ Advantage MAX (Finnigan) mass spectrometer operating in positive ion mode. Proteins were applied onto an Vydac RP-C4 column (Grace) at 20% buffer B (CH_3_CN with 0.08% trifluoroacetic acid) in buffer A (H_2_O plus 0.1% TFA) and eluted with a gradient from 20–80% buffer B at a flow rate of 1 ml min^−1^. Peptide samples were loaded onto the column at 5% buffer B and eluted with a gradient from 5–80% buffer B. Data evaluation was performed with the Xcalibur, MagTran and Bioworks software packages.

## Author contributions

C.A.B. expressed, purified and crystallized the proteins with the help of L.F. and K.V.-B., determined its structure together with K.A. and analysed its kinase activity. C.H. and D.E. determined *in vitro* kinase activity with precipitated CTD, M.M.S. and R.P.F. analysed Cdk12 activation, and D.B. performed kinase activity measurements of flag-tagged proteins. L.F. performed small molecule inhibition experiments. M.G. designed the study and wrote the manuscript with support of D.E. and R.P.F. All authors discussed the results and commented on the manuscript.

## Additional information

**How to cite this article:** Bösken, C. A. *et al*. The structure and substrate specificity of human Cdk12/Cyclin K. *Nat. Commun.* 5:3505 doi: 10.1038/ncomms4505 (2014).

**Accession code:** Atomic coordinates and structure factor files for the Cdk12/CycK complex have been deposited in the Protein Data Bank under accession code 4NST.

## Supplementary Material

Supplementary Information Supplementary Figures 1-11, Supplementary Table 1 and Supplementary References

## Figures and Tables

**Table 1:** Data collection and refinement statistics.

X-ray source	Synchrotron
Beam line	SLS X10SA
Wavelength (Å)	0.9785
Temperature (K)	100
*Crystal information*	
Resolution (Å)†	2.2
Space group	P1
Unit cell dimensions	
a, b, c (Å)	a=49.72, b=78.85 Å, c=91.49 Å
α, β, γ (°)	α=104.04, β=85.76, γ=101.46
*R*_meas_‡	0.079 (0.368)
Mean *I*/σ_I_	12.5 (3.7)
Completeness (%)	97.3 (97.2)
Solvent content (%)	48.1
*Refinement*	
Unique reflections	65,263
No. of reflections used	65,227
Model contents	A: Cdk12 (716–1,046, Δ799–806)B: CycK (20–265)C: Cdk12’ (717–1,045, Δ797–806, Δ887)D: CycK’ (21–261)
*R*_work_/*R*_free_§	0.195/0.240
*No. of atoms*	
Protein	9,215
Water	387
Ligand(s)	86
*B factors (average B value (Å^2^))*	
Protein	42.9
Water	43.1
Ligands	43.3
*R.m.s deviations*	
r.m.s. deviations bonds (Å)	0.015
r.m.s. deviations angles (°)	1.6
Ramachandran plot	most favored: 98.7%, allowed: 1%
PDB accession code	4NST

*R*=Σ_*hkl*_||*F*_obs_| − |*F*_calc_||/Σ|*F*_obs_|, where *F*_obs_ and F_calc_ are the observed and calculated structure factors of reflection *hkl*.

*R*_free_ was calculated from a randomly selected subset of the reflections (5%) that were omitted during refinement.

^†^Values in parentheses correspond to the highest resolution shell (2.25–2.20 Å).

^‡^R_meas_ is the redundancy-independent merging R-factor (intensities). *R*_meas_ =(Σ*h*(*n*/(*n*−1))^0.5^ Σ*j*|*I*^*^*^_*h*_ -*Ihj*|)/(Σ*hjIhj*) with *I*^*^*^_*h*_ =(Σ*jIhj*)/*nj.* Where *N* is the number of times a given observation has been observed (that is, *j*=1,*n*).

^§^5% of the total reflections were excluded for cross-validation.
