# Supplementary material for: The structure and substrate specificity of human Cdk12/Cyclin K
Source: Nat Commun. 2014 Mar 24;5:3505. doi: 10.1038/ncomms4505 (PMC3973122; doi:10.1038/ncomms4505)
Supplement: Supplementary Information — Supplementary Figures 1-11, Supplementary Table 1 and Supplementary References [file ncomms4505-s1.pdf]

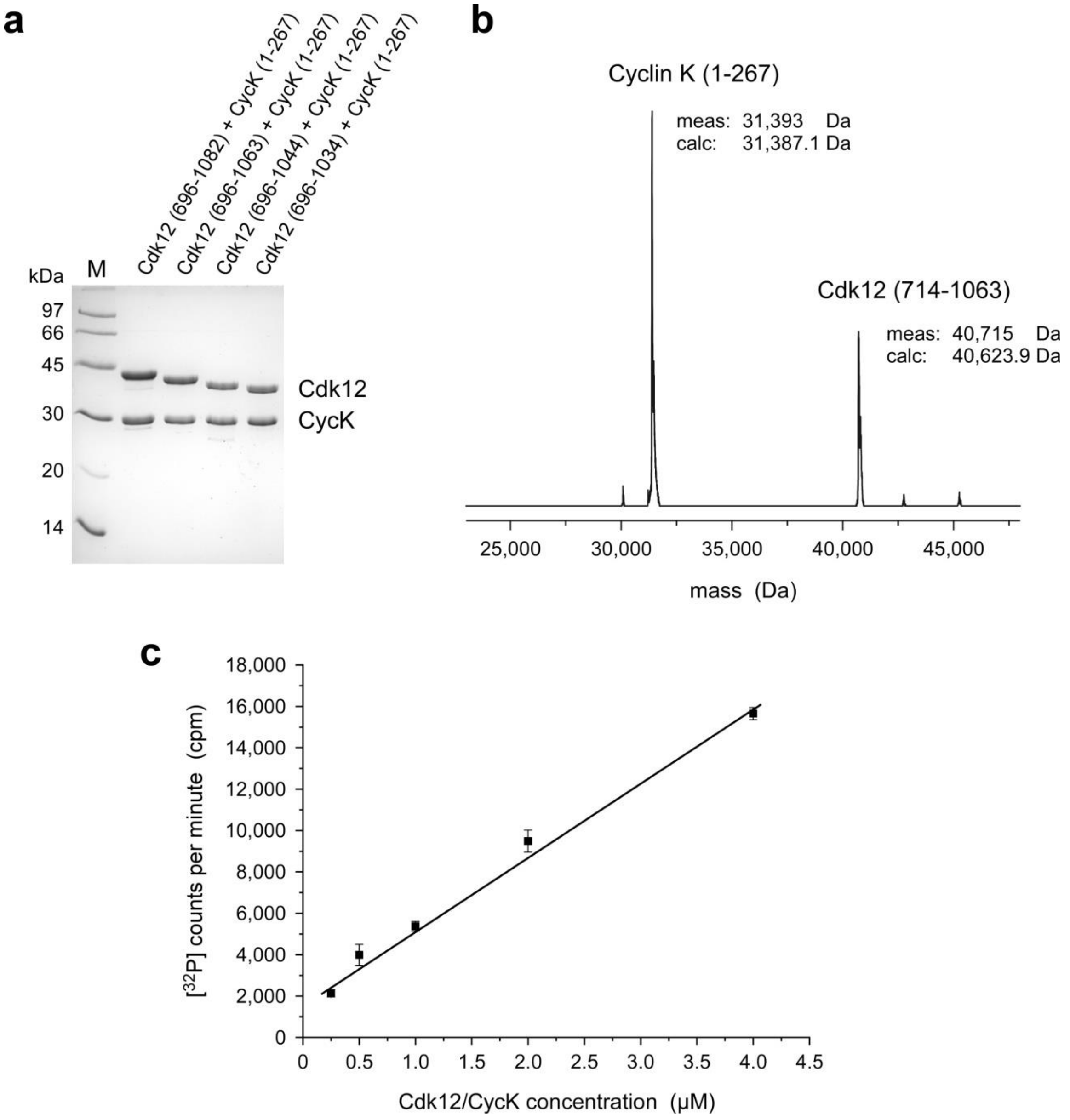

Supplementary Figure 1: Generation and activity of the Cdk12/CycK complex.
(**a**) Coomassie stained SDS PAGE display of various Cdk12/CycK protein complexes. The kinase–cyclin complexes were purified to homogeneity by affinity chromatography and gel filtration.
(**b**) ESI mass spectrometry of purified Cdk12/CycK indicated mono-phosphorylation of Cdk12. The mass of human Cdk12 (714-1063) was calculated to 40,623.9 Da, whereas the determined mass showed an additional +91 Da corresponding to one phosphorylation group in the T-loop as seen in the protein crystal density map (+80 Da) and potentially one additional oxidation.
(**c**) Activity of Cdk12/CycK for CTD substrate phosphorylation. A linear dependency of the kinase activity on the Cdk12/CycK complex concentration was observed using excess CTD-pS7 as substrate and ATP as co-substrate. The activity (in counts per minute) was measured by radioactive labeling of pS7-$CTD_{[3]}$ by transfer from $[^{32}P]$-γ-ATP to the substrate using a filter binding assay. Data are the mean ± s.d. of three independent experiments.

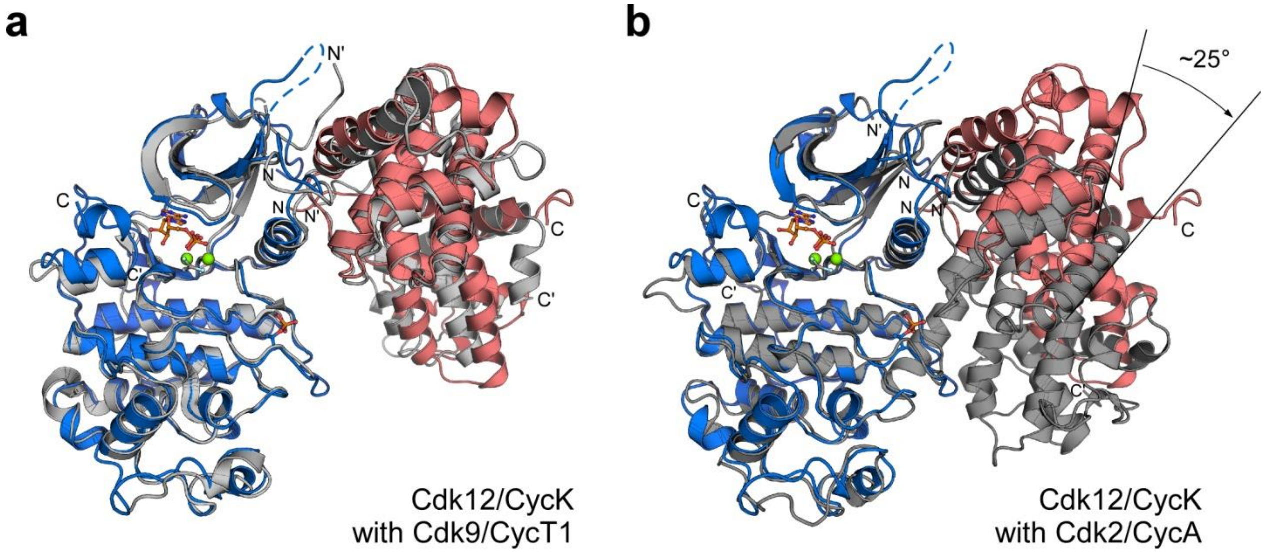

Supplementary Figure 2: Comparison of Cdk/Cyclin structure assemblies.
(**a**) Superimposition of Cdk12/CycK (colored blue/red, 4NST, this study) with Cdk9/CycT1 (grey, 3BLQ)[1]. Both transcription active kinase pairs share a similar orientation of the two subunits.
(**b**) In contrast, overlay of Cdk12/CycK (colored blue/red, 4NST, this study) with Cdk2/CycA (grey, 1JST)[2] by superimposition of the two kinases shows that the orientation of the cyclin with respect to the Cdk subunit is twisted inward by about 25° toward a closer conformation of the cell cycle kinase–cyclin complex.

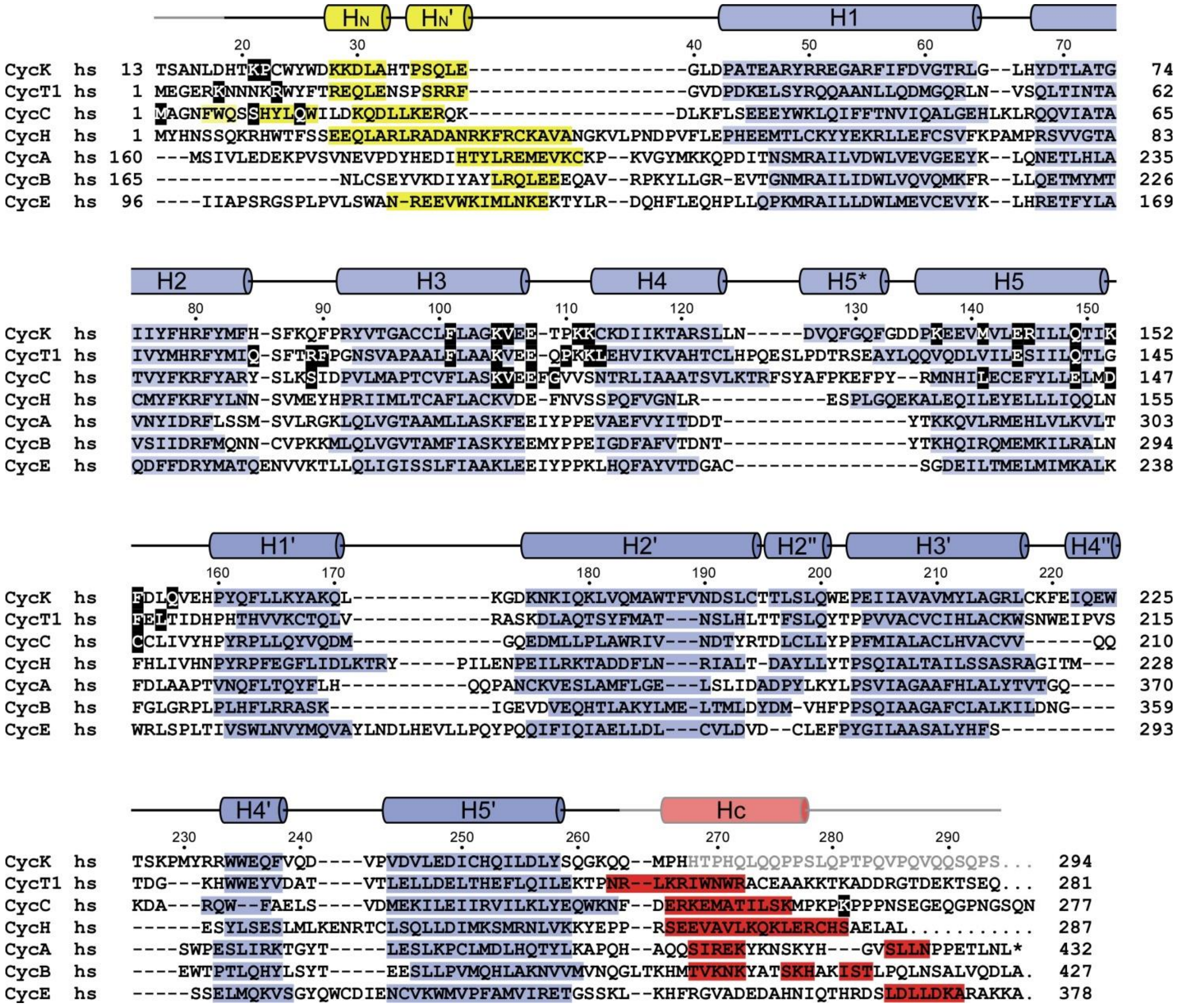

Supplementary Figure 3: Structure-based sequence alignment of cyclin box repeats from various cyclin proteins.

The sequence alignment is based on the structures of human Cyclin K (sequence accession number O75909, residues outside the protein construct used for crystallization are colored grey; this study), human CycT1 (O60563; PDB accession number 2PK2)[3], human Cyclin C (P24863; 3RGF)[4], human Cyclin H (P51946; 1JKW)[5], human Cyclin A (P20248; 1FIN)[6], human Cyclin B (P14635; 2B9R)[7] and human Cyclin E (P24864; 1W98)[8]. Secondary structure elements of human CycK as derived from the complex with Cdk12 are indicated on top. Helices of Cyclins K, T1, C, H, A, B, and E are displayed by bars with the N- and C-terminal helices colored yellow and red, respectively. Residues of CycK that interact with Cdk12 in a distance shell smaller than 3.5 Å are boxed black. Residues of CycT1 and CycC that interact with Cdk9 and Cdk8 within 3.5 Å distance, respectively, (3TNH and 3RGF)[9,4] are similarly marked.

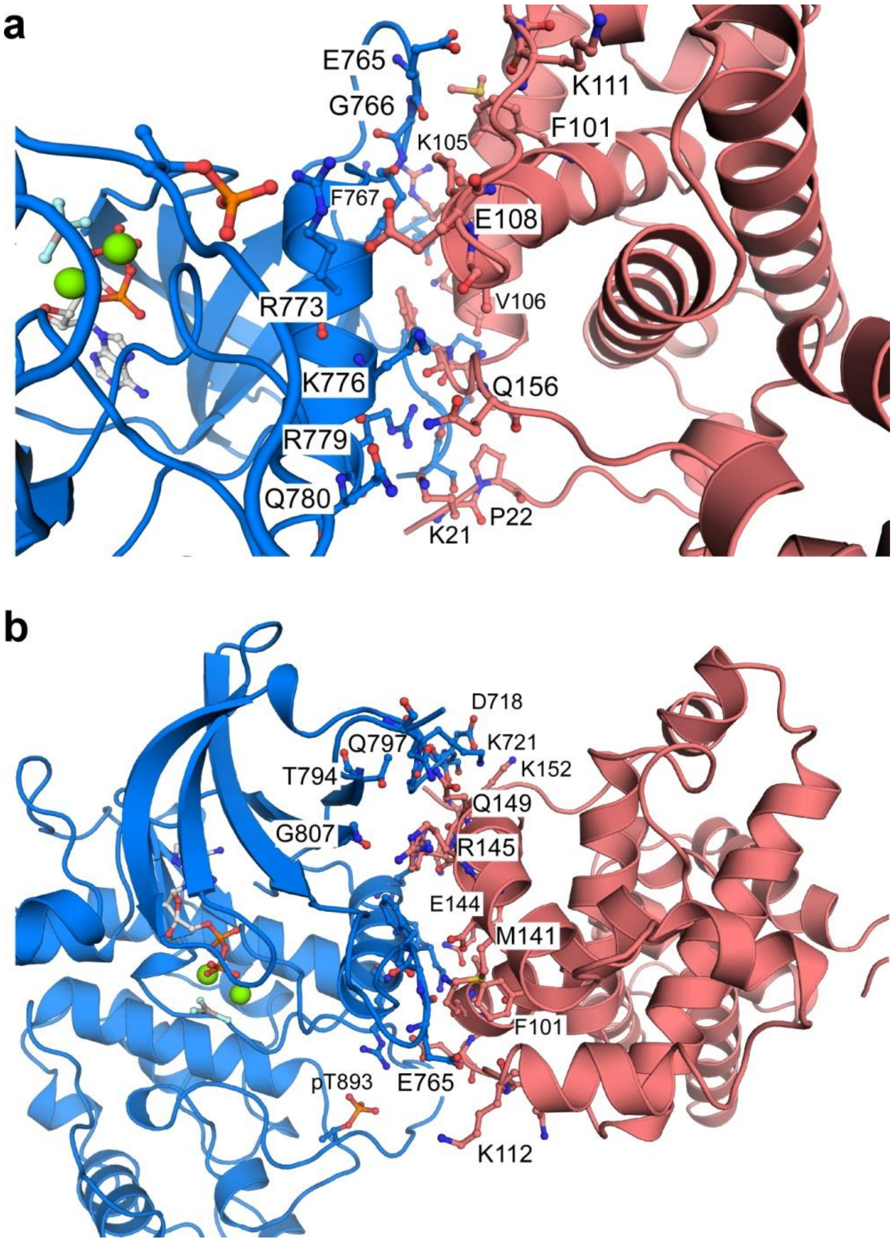

Supplementary Figure 4: Details of the Cdk12–CycK interaction.

(**a**) Residue on helix H3 and the following loop section (F101 to K112) of CycK interact with the N-terminal lobe of Cdk12. N-terminal residues K21 and P22 contribute to the specificity of this interaction.

(**b**) A second interaction site on CycK for Cdk12 is formed by residues on helix H5 and the adjacent loop (M141 to Q156). These residues interact with the N-terminus of the Cdk12 kinase domain (D718, K721) and the central β-sheet of the kinase N-terminal lobe ($R145_{CycK}$ to G807 and T794). In contrast to Cdk2, the entire interaction site of Cdk12 to CycK is formed by residues of its N-terminal lobe only. Nonetheless, the buried surface area on the kinase subunit encompasses 1126 $Å^2$.

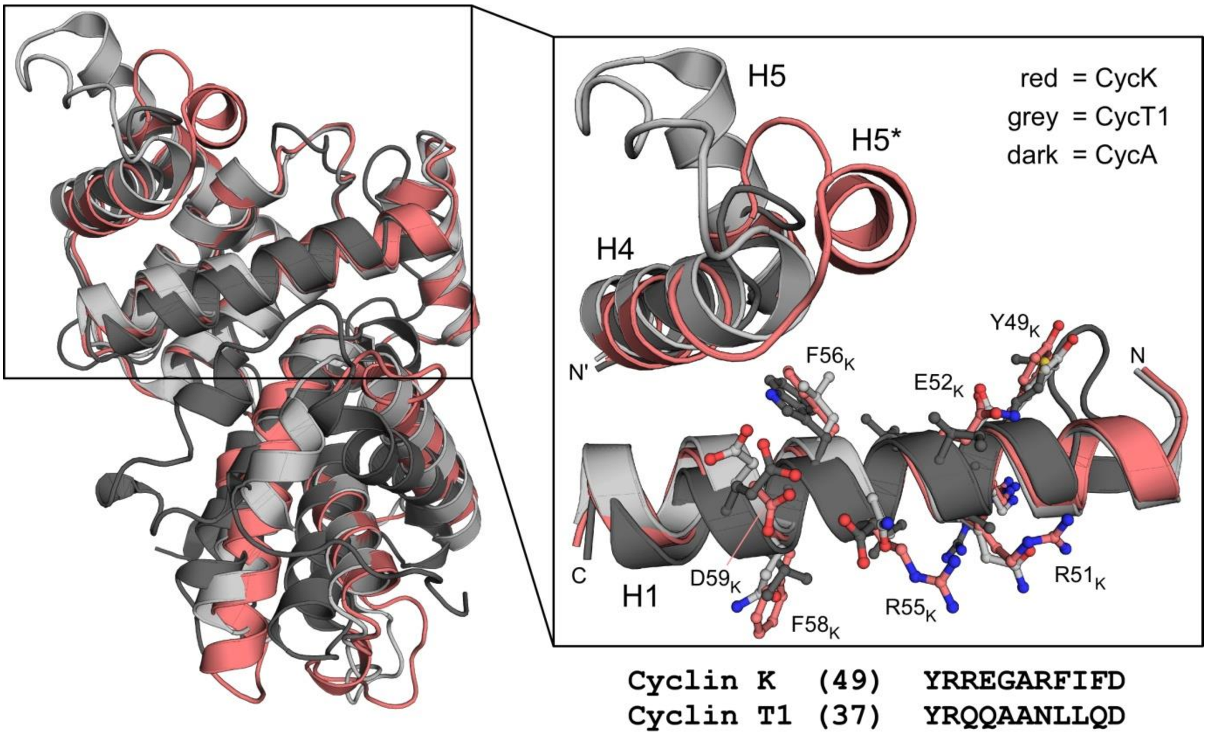


```
Cyclin K  (49)   YRREGARFIFD
Cyclin T1 (37)   YRQQAANLLQD
Cyclin A (210)   MRAILVDWLVE
```

Supplementary Figure 5: **Structural alignment of the MRAILVDWxxE motif in Cyclins A, T1 and K.**

The structure alignment is based on the structures of human CycK (this study), human CycT1 (3BLQ)[1], and human CycA (1JST)[2]. The MRAILVDWxxE motif in CycA, responsible for binding to substrate and inhibitor sequences is replaced with the hydrophilic YRREGARFxxD sequence in CycK. The insertion of 11 residues in CycK or 16 residues in CycT1 compared to CycA (see Supplementary Fig. 3) leads to additional structural elements in between helices H4 and H5 that partly cover the interaction site on helix H1.

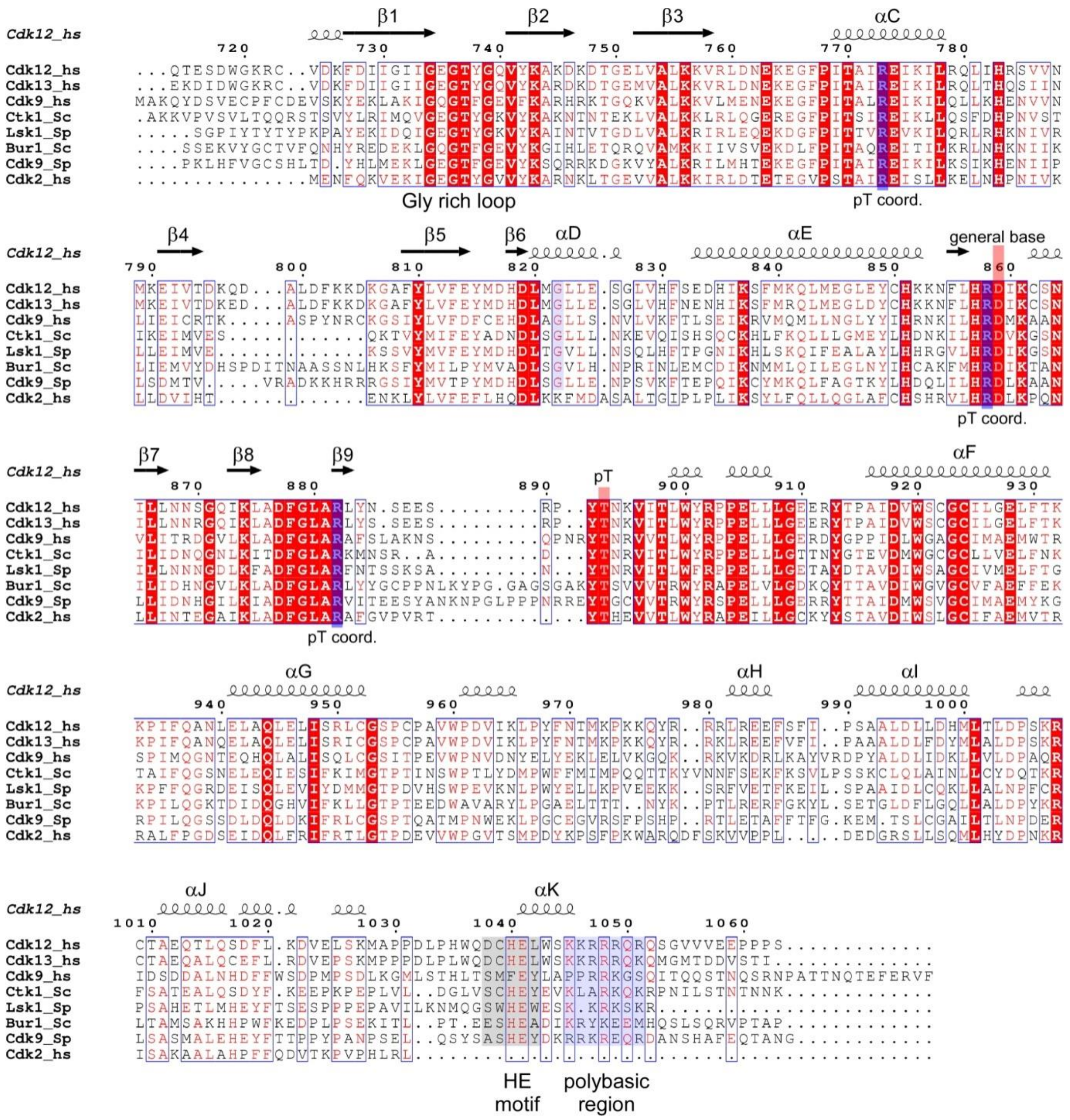

Supplementary Figure 6: Alignment of Cdk sequences.

Sequence alignment of Cdk9 and Cdk12 type kinases from human, *S. cerevisiae* and *S. pombe*, compared to human Cdk2. Secondary structure elements are indicated for Cdk12 as determined here. Residues conserved in all CDKs are boxed red and those that are similar are colored red. The newly identified HE motif with the adjacent polybasic cluster at the C-terminus is highlighted. The presence of these motifs correlates with a glycine residue in helix αD (G822) representing potentially a specific feature of CTD kinases. The UniProt accession numbers are: Q9NYV4 (Cdk12 human), Q14004 (Cdk13 human), P50750 (Cdk9 human), P23293 (Bur1, *S.c.*), Q03957 (Ctk1, *S.c.*), Q96WV9 (Cdk9, *S.p.*), O14098 (Lsk1, *S.p.*) and P24941 (Cdk2, human). The sequence alignment was prepared with MultAlin, and the secondary structure alignment was prepared with ESPript.

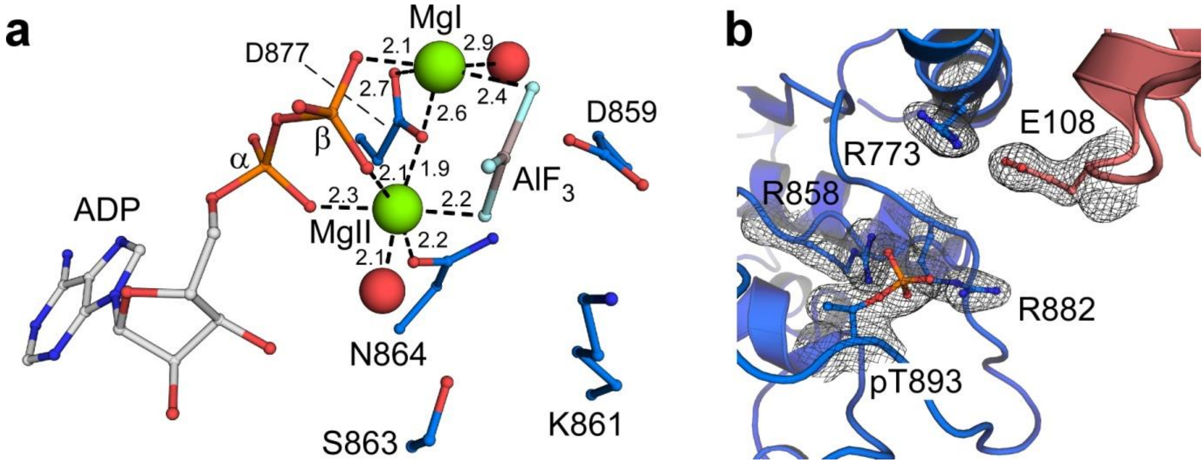

Supplementary Figure 7: Structural details of active Cdk12.

(**a**) Coordination environment of the two magnesium ions. Distances to the α- and β-phosphate oxygens, N864, the two oxygens of D877 (DFG motif), two water molecules, and AlF_3 as the γ-phosphate mimic are displayed.

(**b**) Close-up of pT893 in the Cdk12 T-loop segment and side chain contacts. R773 of the P_{768}ITAIRE sequence forms electrostatic contacts with E108 of the K_{105}VEE motif but is not directly contacting pT893. The other two canonical arginines, R858 and R882, form ionic interactions with the phosphate group. The final $2F_o$–F_c electron density is displayed at 1σ.

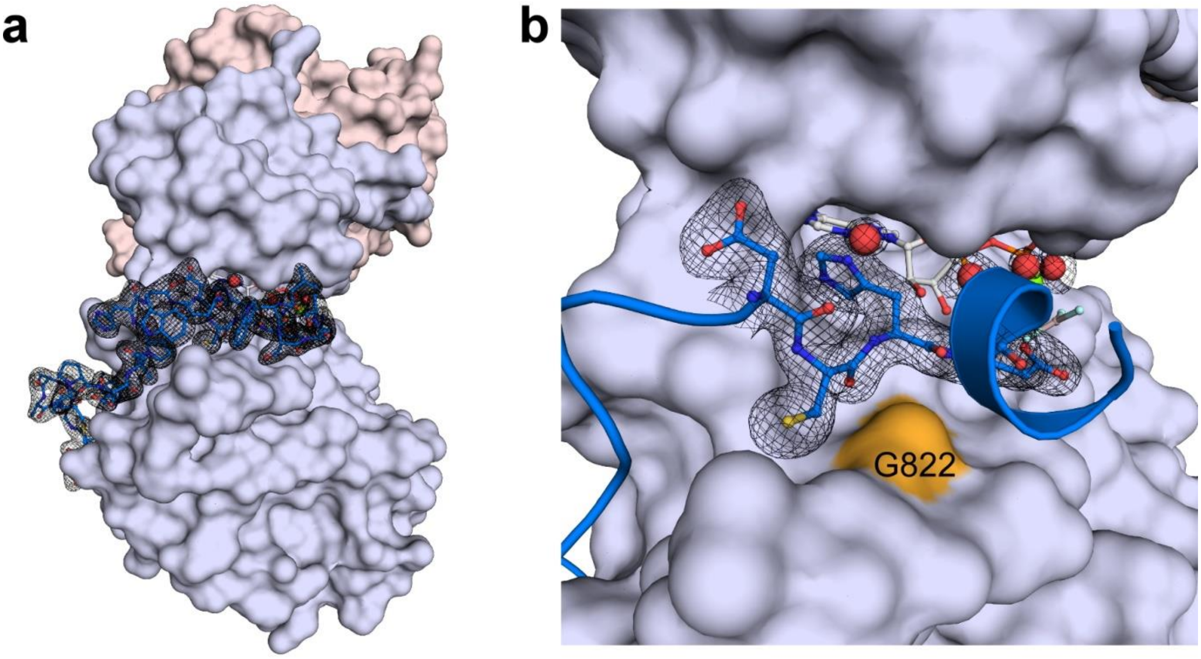

Supplementary Figure 8: The C-terminal extension of Cdk12 associates with the ATP co-substrate.

(**a**) Coordination of the C-terminal extension in Cdk12 following the canonical kinase domain. Residues 1022–1046 are shown as cartoon with the final $2F_o$–F_c electron density displayed at 1 σ. The Cdk12/CycK core structure is displayed as surface representation.

(**b**) Assembly of the C-terminal helix in Cdk12 with the N- and C-terminal lobe and the adenine nucleotide. The final $2F_o$–F_c electron density of the interacting residues (DCHEL) in the HE-motif as well as water molecules that mediate contacts to the bound nucleotide are displayed at 1 σ. The position of residue G822 that generates space for the association of the C-terminal helix with the nucleotide is highlighted orange on the surface of Cdk12.

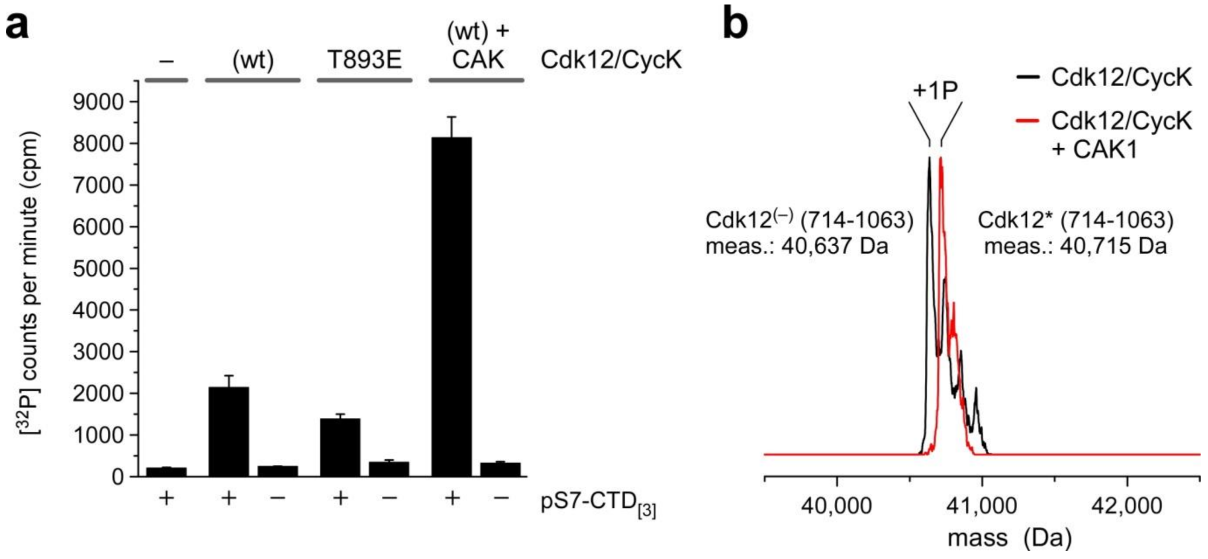

Supplementary Figure 9: T-loop phosphorylation of Cdk12 by Cak1.
(**a**) Coexpression of Cdk12/CycK with Cak1 from *S. cerevisiae* leads to kinase T-loop activation. T-loop non-phosphorylated $Cdk12^{(-)}$/CycK (wt) shows some residual activity for $pS7\text{-}CTD_{[3]}$ substrate phosphorylation. Mutation T893E as a phosphoryl group mimic does not increase kinase activity, while co-expression with Cak1 increases the Cdk12 activity fourfold. Data are the mean ± s.d. of three independent experiments.
(**b**) ESI mass spectrometry analysis of Cdk12 (714-1063) co-expressed with Cak1 indicates a mass increase of ~80 Da, corresponding to one additional phosphorylation as confirmed in the crystal structure.

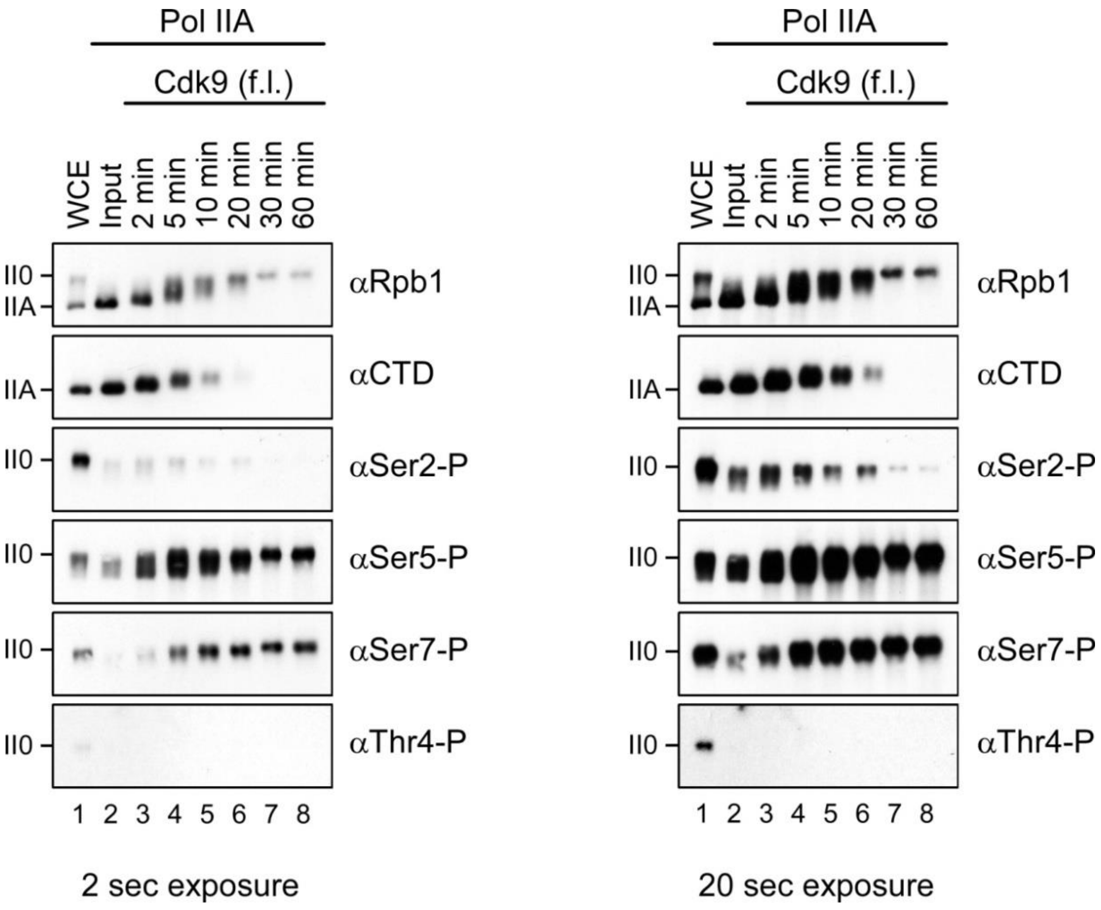

Supplementary Figure 10: Time course of P-TEFb activity and phosphorylation specificity on RNA Pol II Rpb1.

Restriction of epitope recognition by mAbs. Pol II was immuno-precipitated with antibody 1C7 from HeLa cell extracts, incubated with Cdk9/CycT1 (0.5 µg) at various time periods and analyzed by Western blot. Monoclonal antibodies specific for CTD modifications Ser2-P (3E10), Ser5-P (3E8), Ser7-P (4E12), and Thr4-P (6D7), or Rpb1 (Pol3.3.) and non-modified CTD (1C7, (αCTD)) were used as primary antibodies. Cdk9 exhibits the highest activity for Ser5 phosphorylation on the CTD followed by Ser7 phosphorylation. Note that the recognition of the CTD decreases (second panel) as the phosphorylation status of the CTD increases due to the specificity of the antibody against the non-phosphorylated hepta-sequence. Recognition of CTD by Ser2-P mAb 3E10 and 1C7 mAb is inhibited with increase of Ser7-P and/or Ser5-P signals. The hyper- (II0) and hypo-phosphorylated (IIA) forms of RNA Pol II are indicated, with the hypo-phosphorylated form running at an aberrant molecular mass of 250 kDa. Shown is the same panel of Western blots left and right with 2 and 20 s exposure times.

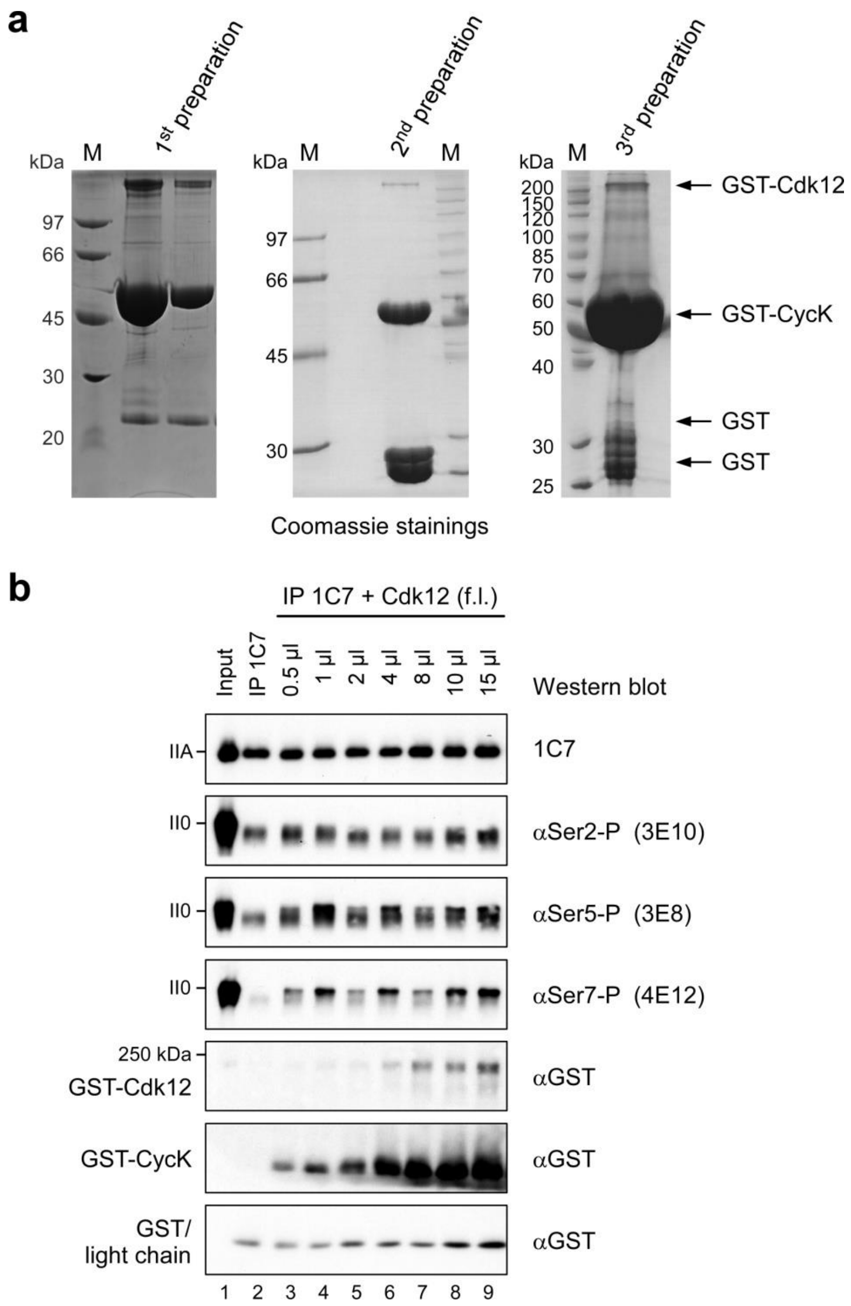

Supplementary Figure 11: Purification and activity of recombinant full length Cdk12. (**a**) Coomassie stained SDS PAGE analysis of purified human full length Cdk12 (1-1490) as a GST fusion protein. Cdk12 (1-1490) was expressed in baculo virus infected *Sf21* insect cells together with Cyclin K and CAK1. Three independent expression and purification procedures were performed. The identity of GST-Cdk12 at ~220 kDa was confirmed by peptide mass finger print analysis. Due to higher expression levels of CycK, stoichiometric levels of the Cdk12/CycK complex could not be achieved during the purification procedure.
(**b**) Pol II Rpb1 was immuno-precipitated with antibody 1C7 from HeLa whole cell extracts, incubated with increasing concentrations of full length Cdk12/CycK and displayed by Western blot analysis. Monoclonal antibodies specific for CTD modifications Ser2-P (3E10), Ser5-P (3E8), and Ser7-P (4E12), or non-modified CTD (1C7) were used as primary antibodies. αGST antibodies were used as input control (lower three blots). No significant increases in the phosphorylation status of Ser2, Ser5 or Ser7 in the precipitated CTD could be observed suggesting that the recombinant full length kinase is largely inactive.

Supplementary Table 1. Overview of CTD peptides used in this study

CTD peptides	Sequence[a]	calculated mass (Da)
cons. CTD[3]	YSPTSPS YSPTSPS YSPTSPS-[PEG2]-RR-amid	2633.8
pY1-CTD[3]	PS **pY**SPTSPS **pY**SPTSPS **pY**SPTSPS-[PEG2]-RR-amid	3056.0
pS2-CTD[3]	S Y**pS**PTSPS Y**pS**PTSPS Y**pS**PTSPS-[PEG2]-RR-amid	2960.8
pT4-CTD[3]	S YSP**pT**SPS YSP**pT**SPS YSP**pT**SPSY-[PEG2]-RR-amid	3124.0
pS5-CTD[3]	YSPT**pS**PS YSPT**pS**PS YSPT**pS**PS-[PEG2]-RR-amid	2873.8
pS7-CTD[3]	ac-YSPTSP**pS** YSPTSP**pS** YSPTSP**pS**Y-[PEG2]-RR-amid	3079.0
K7-CTD[3]	YSPTSP**K** YSPTSP**K** YSPTSP**K**-[PEG2]-RR-amid	2757.1
S2A CTD[3]	S Y**A**PTSPS Y**A**PTSPS Y**A**PTSPS-[PEG2]-RR-amid	2672.9
S5A CTD[3]	YSPT**A**PS YSPT**A**PS YSPT**A**PS-[PEG2]-RR-amid	2585.8
S2A_pS7 CTD[3]	Y**A**PTSP**pS** Y**A**PTSP**pS** Y**A**PTSP**pS**-[PEG2]-RR-amid	2825.8
S5A_PS7 CTD[3]	YSPT**A**P**pS** YSPT**A**P**pS** YSPT**A**P**pS**Y-[PEG2]-RR-amid	2989.0
pS2-N CTD[3]	ac-Y**pS**PTSPS YSPTSPS YSPTSPS-[PEG2]-RR-amid	2755.9
pS2-C CTD[3]	ac-SYSPTSPS YSPTSPS Y**pS**PTSPS-[PEG2]-RR-amid	2842.9
pS5-N CTD[3]	YSPT**pS**PS YSPTSPS YSPTSPS-[PEG2]-RR-amid	2713.9
pS5-C CTD[3]	S YSPTSPS YSPTSPS YSPT**pS**PS-[PEG2]-RR-amid	2800.9
pS7-N CTD[3]	TSP**pS** YSPTSPS YSPTSPS YSPT-[PEG2]-RR-amid	2856.9
pS7-C CTD[3]	YSPTSPS YSPTSPS YSPTSP**pS** YS-[PEG2]-RR-amid	2964.1
S5_pS7_13mer CTD	ac-P**pS** YSPTSP**pS** YSPT-amid	1571.4

[a] Peptides used for the quantitative determination of phosphorylation numbers were marked with a double polyethylene glycol linker as a spacer and two arginines for better ionization properties in the ESI MS analysis.

Supplementary References

1. Baumli, S. *et al.* The structure of P-TEFb (CDK9/cyclin T1), its complex with flavopiridol and regulation by phosphorylation. *EMBO J.* **27**, 1907–1918 (2008).
2. Russo, A.A., Jeffrey, P.D. & Pavletich, N.P. Structural basis of cyclin-dependent kinase activation by phosphorylation. *Nat. Struct. Biol.* **3**, 696–700 (1996).
3. Anand, K., Schulte, A., Fujinaga, K., Scheffzek, K. & Geyer, M. Cyclin box structure of the P-TEFb subunit Cyclin T1 derived from a fusion complex with EIAV Tat. *J. Mol. Biol.* **370**, 826–836 (2007).
4. Schneider, E.V. *et al.* The structure of CDK8/CycC implicates specificity in the CDK/cyclin family and reveals interaction with a deep pocket binder. *J. Mol. Biol.* **412**, 251–266 (2011).
5. Andersen, G. *et al.* The structure of cyclin H: common mode of kinase activation and specific features. *EMBO J.* **16**, 958–967 (1997).
6. Jeffrey, P.D. *et al.* Mechanism of CDK activation revealed by the structure of a cyclinA-CDK2 complex. *Nature* **376**, 313–320 (1995).
7. Petri, E.T., Errico, A., Escobedo, L., Hunt, T. & Basavappa, R. The crystal structure of human Cyclin B. *Cell Cycle* **6**, 1342–1349 (2007).
8. Honda, R. *et al.* The structure of cyclin E1/CDK2: implications for CDK2 activation and CDK2-independent roles. *EMBO J*. **24**, 452–463 (2005).
9. Baumli, S., Hole, A.J., Noble, M.E. & Endicott, J.A. The CDK9 C-helix exhibits conformational plasticity that may explain the selectivity of CAN508. *ACS Chem. Biol.* **7**, 811–816 (2012).
